# PELI1: key players in the oncogenic characteristics of pancreatic Cancer

**DOI:** 10.1186/s13046-024-03008-9

**Published:** 2024-03-25

**Authors:** Xiaobin Fei, Changhao Zhu, Peng Liu, Songbai Liu, Likun Ren, Rishang Lu, Junyi Hou, Yongjia Gao, Xing Wang, Yaozhen Pan

**Affiliations:** 1 School of Clinical Medicine, Guizhou Medical University, Guiyang, Guizhou Province China; 2 Department of Hepatobiliary Surgery, Affiliated Cancer Hospital of Guizhou Medical University, Guiyang, Guizhou Province China; 3 Department of Hepatobiliary Surgery, Affiliated Hospital of Guizhou Medical University, Guiyang, Guizhou Province China; 4 Department of Hepatobiliary Surgery, Baiyun Hospital of Guizhou Medical University, Guiyang, Guizhou Province China

**Keywords:** Pancreatic cancer, PELI1, RPS3, PI3K/Akt/GSK3β, p53

## Abstract

**Background:**

Pancreatic cancer (PC) is a highly malignant gastrointestinal tumor, which is characterized by difficulties in early diagnosis, early metastasis, limited therapeutic response and a grim prognosis. Therefore, it is imperative to explore potential therapeutic targets for PC. Currently, although the involvement of the Pellino E3 Ubiquitin Protein Ligase 1 (PELI1) in the human growth of some malignant tumors has been demonstrated, its association with PC remains uncertain.

**Methods:**

Bioinformatics, qRT-PCR, Western blot and IHC were used to detect the expression of PELI1 in pancreas or PC tissues and cells at mRNA and protein levels. The effects of PELI1 on the proliferation and metastatic ability of pancreatic cancer in vitro and in vivo were investigated using CCK8, cloning formation, EdU, flow cytometry, IHC, Transwell assay, wound healing, nude mice subcutaneous tumorigenesis and intrasplenic injection to construct a liver metastasis model. The interactions of PELI1 with proteins as well as the main functions and pathways were investigated by protein profiling, Co-IP, GST-pull down, Immunofluorescence techniques, immunohistochemical co-localization and enrichment analysis. The rescue experiment verified the above experimental results.

**Results:**

The mRNA and protein expression levels of PELI1 in PC tissues were upregulated and were associated with poor prognosis of patients, in vitro and in vivo experiments confirmed that PELI1 can affect the proliferation and metastatic ability of PC cells. Co-IP, GST-pull down, and other experiments found that PELI1 interacted with Ribosomal Protein S3 (RPS3) through the FHA structural domain and promoted the polyubiquitination of RPS3 in the K48 chain, thereby activates the PI3K/Akt/GSK3β signaling pathway. Moreover, ubiquitinated degradation of RPS3 further reduces Tumor Protein P53 (p53) protein stability and increases p53 degradation by MDM2 Proto-Oncogene (MDM2).

**Conclusion:**

PELI1 is overexpressed in PC, which increased ubiquitination of RPS3 proteins and activates the PI3K/Akt/GSK3β signaling pathway, as well as reduces the protective effect of RPS3 on p53 and promotes the degradation of the p53 protein, which facilitates the progression of PC and leads to a poor prognosis for patients. Therefore, PELI1 is a potential target for the treatment of PC.

**Supplementary Information:**

The online version contains supplementary material available at 10.1186/s13046-024-03008-9.

## Introduction

Pancreatic cancer (PC), a common malignancy in the gastrointestinal system, presents challenges in early diagnosis and treatment, resulting in a less than 10% 5-year survival rate and a grim prognosis [1]. PC incidence increases annually [2], elevating its rank from seventh to third in terms of human cancer mortality [1, 3]. Pancreatic ductal adenocarcinoma (PDAC) constitutes over 90% of all pancreatic malignancies [4]. Currently, the most effective and exclusive opportunity for curing resectable and borderline resectable PC is radical resection, combined with perioperative systemic treatment [5]. However, due to the lack of typical clinical symptoms and effective screening tools, approximately 80–85% of patients already lost the opportunity for radical surgical treatment upon PC diagnosis [6]. Owing to PC’s biological characteristics of high aggressiveness, early metastasis, and susceptibility to treatment resistance [7, 8], even after surgical removal of the lesion or a combination of medication treatment, most patients remain at risk of recurrence [9]. Therefore, the current emphasis in cancer investigation is to clarify the essential molecular mechanisms and communication pathways contributing to the advancement of PC. This pursuit aims to provide a theoretical foundation and offer practical insights for the timely detection and treatment of PC.

The ubiquitin-proteasome system (UPS) constitutes a complex process of post-translational protein modification by ubiquitinating and deubiquitinating enzymes, jointly controlling protein biological activity [10, 11]. It affects almost every function of eukaryotic cells, including transcription, cell proliferation, apoptosis, cell cycle progression, inflammation, etc. [12]. E3 ubiquitin ligase, a key player in the UPS, determines the recognition specificity of ubiquitination for most substrates [13, 14]. Thus, E3 ubiquitin ligases play a crucial role in maintaining eukaryotic cellular homeostasis. Abnormal expression and dysfunction of E3 ubiquitin ligase have been linked to various human diseases, including the initiation and advancement of cancer [15–17].

PELI1, a member of the Pellino family of E3 ubiquitin ligases, plays a significant role in various biological processes. These functions encompass inducing necrotic apoptosis, stimulating T-cells, mediating inflammatory responses, and involvement in immunological disorders, among other tasks [18–21]. Crucially, aberrant PELI1 expression strongly correlates with the development of solid tumors. It synergizes with EGFR to promote breast cancer metastasis [22], thyroid cancer progression [23], also can enhance esophageal cancer radiotherapy sensitivity [24]. Despite PELI1’s well-established cancer-promoting role in solid tumors, its exploration in PC remains uncharted. Unraveling the mechanisms underlying PELI1’s actions could potentially contribute to the treatment of PC.

Ribosomal protein S3 (RPS3), a protein with multiple extra-ribosomal functions involved in DNA damage repair and apoptosis [25], which is regulated through various post-translational modifications, including methylation, ubiquitination, and acetylation [26, 27]. Imai Ayaka et al. suggested that salicylic acid binds to RPS3, hindering the cell cycle progression of colon cancer cells [28]. Additionally, ubiquitinated degradation of RPS3 is crucial for the emergence of radiation resistance in glioblastoma [29], underscoring its major role in initiating apoptosis in glioblastoma. The current state of RPS3 study in PC is nascent, and the corresponding cause-and-effect relationship remains unclear. Understanding RPS3’s function in PC could potentially present a new target for therapeutic intervention.

This study revealed that elevated PELI1 expression is associated with a poor prognosis for PC patients and is highly expressed in PC cells. Demonstrations highlighted that PELI1 promotes the malignant biological behaviour of PC both in vivo and in vitro. PELI1 facilitates RPS3 degradation through polyubiquitination on the K48 chain by interacting with RPS3 through the FHA structural domain. This interaction not only activates the PI3K/Akt/GSK3β signaling pathway but also promotes the ubiquitination and degradation of p53 by MDM2, thereby enhancing PC cell proliferation and metastasis. The present study identified the regulatory mechanism of PELI1-mediated RPS3 in PC, offering a potential new target for treatment of PC.

## Result

### PELI1 expression in pancreatic Cancer

To confirm the expression of PELI1 at the transcriptional and translational levels in pancreatic cancer tissues and cells, we analyzed mRNA expression in normal, PC and paracancerous tissues from the TCGA database, coupled with the GTEx database, revealed a significantly elevated PELI1 mRNA expression in PC compared to normal or paracancerous tissues (Fig. S1A). Data from three distinct sets of PC samples (GSE62165, GSE15471, and GSE71989) available in the GEO database further confirmed a markedly increased expression of PELI1 in PC tissues (Fig. S1B). Notably, the PELI1 high-expression group exhibited worse overall survival (OS), progression-free survival (PFS), and disease-specific survival (DSS) in the TCGA PC survival study (Supplementary Fig. 1C). ROC curve analysis underscored PELI1’s potential as a robust predictor for clinical outcomes in PC (Fig. S1D).

**Fig. 1:**
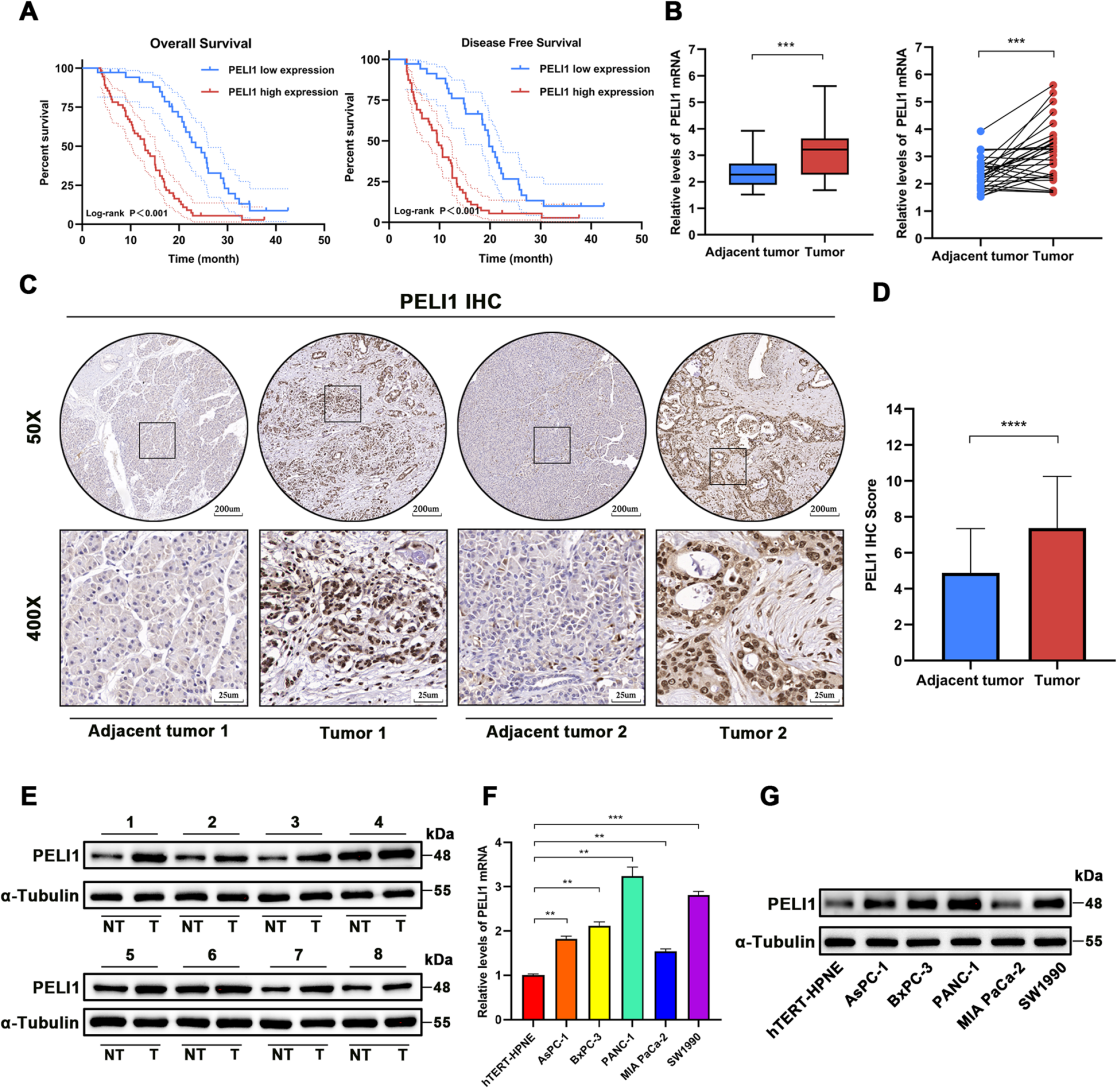
PELI1 Overexpression in PC Tissues and Cell Lines. **A** High PELI1 expression associated with poor prognosis in clinical PC patients. **B**, **C** qRT-PCR (B) and IHC (C) to detect PELI1 expression in PC and paracancerous tissues. **D** IHC scores of PELI1 in PC and paracancerous tissues. **E **Western blot assay for PELI1 protein expression in PC and paracancerous tissues. **F**, **G** qRT-PCR (F) and Western blot (G) to detect PELI1 expression in human normal pancreatic ductal epithelial cells hTERT-HPNE and PC cell lines. ***P < 0.001, ****P < 0.0001

Log-rank tests demonstrated that PC patients in the PELI1 high-expression group experienced shorter OS and DSS times (Fig. 1A). Subsequent analysis of clinicopathological characteristics revealed the association between PELI1 expression level and PC tumor size, pTNM stage, T stage, lymphatic metastasis, distant metastasis, vascular invasion, degree of tumor differentiation, and CA19–9 (Table 1). Factors influencing the prognosis of PC patients included gender, tumor size, pTNM stage, T stage, lymphatic metastasis, distant metastasis, vascular invasion, and PELI1 immunohistochemistry (IHC) staining score. Cox’s risk-proportional regression model identified gender, distant metastasis, and PELI1 IHC score as independent risk factors affecting the prognosis of PC (Table 2), highlighting the connection between PELI1 expression in PC and poor patient prognosis.

**Table 1:** Relationship between PELI1 and clinicopathological characteristics of PC patients (chi-square test)

Clinicopathological features		PELI1 Expression Level		X^2^	P
		Low	High		
Total cases		36	54		
Age	< 60	20	27	0.267	0.605
	≥ 60	16	27		
Gender	Male	24	29	1.499	0.221
	Female	12	25		
Tumor size	< 5	30	30	7.50	0.006
	≥ 5	6	24		
pTNM stage	I-II	34	19	31.331	< 0.001
	III-IV	2	35		
T stage	I-II	26	19	11.852	0.001
	III-IV	10	35		
Lymphatic node metastasis	N0	20	13	9.219	0.002
	N1	16	41		
Distantly metastasized	M0	34	32	13.674	< 0.001
	M1	2	22		
Number of foci	Single	29	40	0.507	0.476
	Multiple	7	14		
Vascular invasion	No	26	26	5.132	0.023
	Yes	10	28		
Nerve violation	No	12	19	0.033	0.856
	Yes	24	35		
Degree of differentiation	Low	8	27	8.42	0.015
	Middle	23	25		
	High	5	2		
CEA		2.375(1.775 ~ 4.305)	3.575(2.375 ~ 2.288)	2.949	0.085
CA19–9		85.6 (24.68 ~ 284.25)	295.95(72.79 ~ 580.70)	6.54	0.011

**Table 2:** Univariate (Log-rank test) and multivariate (Cox risk-proportional model) analyses of clinical characteristics and the association between PELI1 and prognosis of PC

Parameter		Univariate analysis			Multivariate analysis		
		HR	95%CI	P	HR	95%CI	P
Age	< 60	18.53	15.13–21.92	0.223			
	≥ 60	16.88	14.60–19.17				
Gender	Male	19.93	17.15–22.71	0.005			
	Female	14.29	11.77–16.81		1.601	0.95–2.69	0.007
Tumor size	< 5	20.20	17.87–22.51	0.001			
	≥ 5	12.31	9.25–15.37		0.833	0.46–1.52	0.55
pTNM stage	I-II	23.01	20.54–25.48	< 0.001			
	III-IV	10.27	8.62–11.92		2.09	0.97 ~ 4.49	0.06
T stage	I-II	22.12	19.10–25.13	<0.001			
	III-IV	13.21	11.12–15.30		1.52	0.78 ~ 2.96	0.22
Lymphatic node metastasis	N0	23.56	19.56–22.80	< 0.001			
	N1	14.53	12.62–16.45		1.07	0.57 ~ 2.00	0.841
Distantly metastasized	M0	20.89	18.60–23.18	< 0.001			
	M1	9.03	7.23–10.82		3.168	1.64 ~ 6.12	0.001
Number of foci	Single	18.03	15.55–20.50	0.572			
	Multiple	16.82	13.11–20.54				
Vascular invasion	No	19.55	16.63–22.47	0.035			
	Yes	15.13	12.60–17.65		0.92	0.55 ~ 1.56	0.761
Nerve violation	No	18.44	14.60–22.29	0.508			
	Yes	17.13	14.88–19.39				
Degree of differentiation	Low	16.143	13.23–19.06	0.341			
	Middle	18.944	15.60–21.89				
	High	15.214	11.40–19.03				
PELI1 IHC Score		1.41	1.27–1.56	< 0.001	1.24	1.08–1.42	0.002
CEA		1.007	0.99–1.01	0.08			
CA19–9		1.00	1.00–1.001	0.497			

Using qRT-PCR of 30 PC and paracarcinoma samples, we observed significantly higher expression of PELI1 mRNA in the PC samples (Fig. 1B). IHC analysis of 90 paraffin-embedded PC and paracancerous tissues revealed a substantially greater positive rate of PELI1 in cancer tissues than in matching paracancerous tissues (Fig. 1C-D). Western blot further affirmed elevated PELI1 protein expression in PC tissues (Fig. 1E). Notably, PELI1 mRNA and protein levels in human normal pancreatic ductal epithelial cells (hTERT-HPNE) were markedly lower than those in five human PC cell lines (AsPC-1, BxPC-3, PANC-1, MIA PaCa-2, SW1990), as confirmed by qRT-PCR and Western blot analysis (Fig. 1F-G).

### PELI1 impact on PC cell behaviour in vitro

**Fig. 2:**
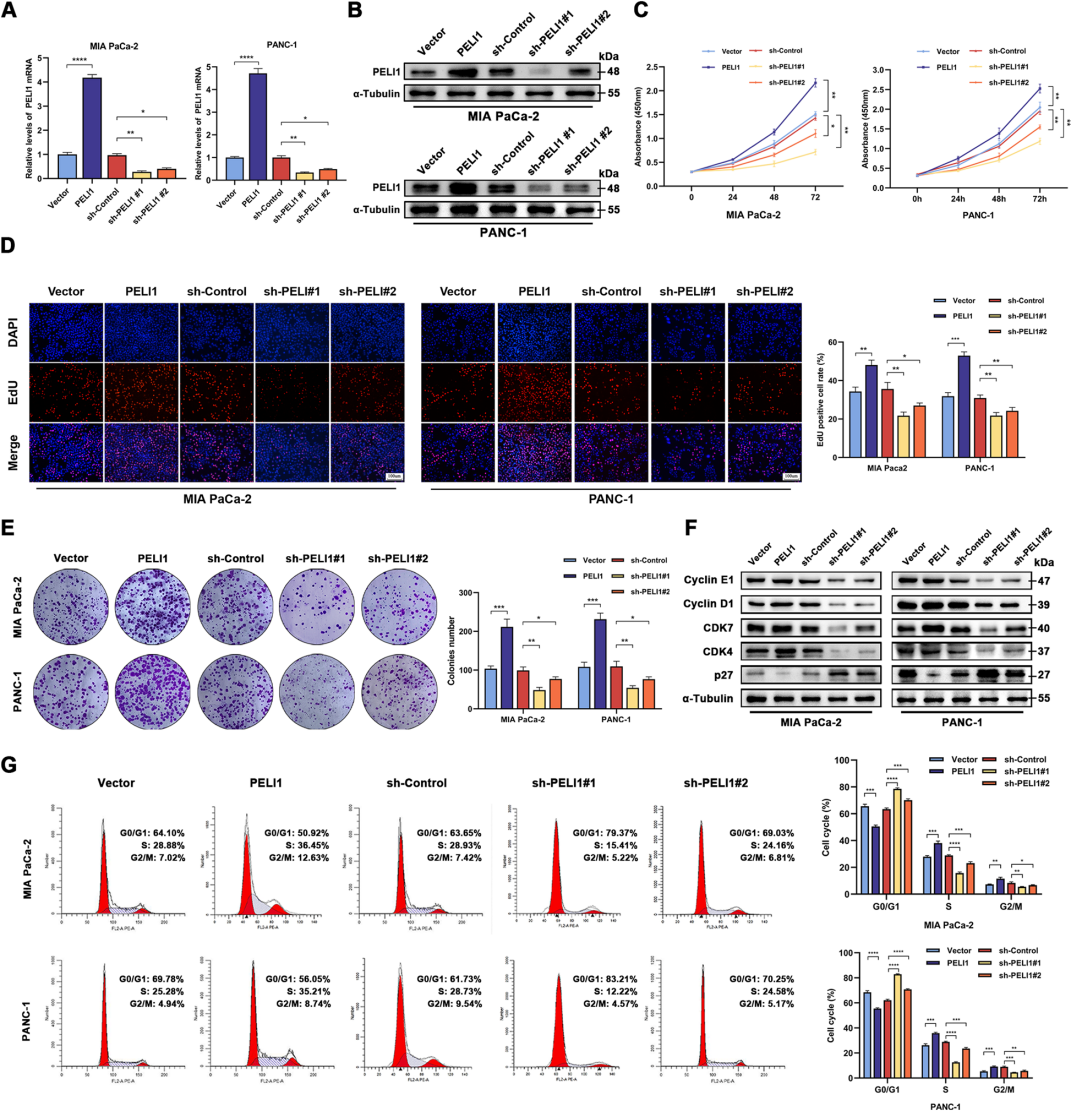
PELI1 Promotes the Proliferation of PC Cells In Vitro. **A**, **B** qRT-PCR (A) and Western blot (B) assay for the construction of stably transfected overexpressing and knockdown PELI1 PC cells. **C**, **D**, **E** CCK-8 (C), EdU (D), and clone formation (**E**) assay for the effect of PELI1 on PC cells proliferative capacity. **F**, **G** Western blot (**F**) and flow cytometry (**G**) to detect the effect of PELI1 on the cell cycle of PC cells. *P < 0.05, **P < 0.01, ***P < 0.001, ****P < 0.0001

PELI1 demonstrates a heightened expression in PC tissues and cells, correlating with a poor prognosis in PC patients. Drawing from this background, we posit a potential pro-cancer role for PELI1 in PC. To substantiate this hypothesis, we selected the MIA PaCa-2 and PANC-1 PC cell lines, relatively low and high PELI1 expression, as experimental subjects. Stable PELI1 overexpression, knockdown, and blank control cell lines with corresponding lentiviral vectors were established, confirming successful construction through qRT-PCR and Western blot analyses (Fig. 2A-B).

CCK8 assays revealed a significant enhancement in the viability of PC cells overexpressing PELI1, contrasting with decreased cell viability upon PELI1 down-regulation (Fig. 2C). EdU staining and clone formation tests further corroborated the augmented cell proliferation ability with PELI1 overexpression and its attenuation with PELI1 down-regulation (Fig. 2D-E). Western blot and flow cytometry experiments demonstrated that PC cells overexpressing PELI1 exhibited accelerated cell cycle progression, while PELI1 down-regulation resulted in inhibited cell cycle advancement (Fig. 2F-G).

Wound healing and Transwell assays affirmed the enhanced migratory and invasive capacities of PELI1 overexpression PC cells, in contrast to decreased invasive abilities upon PELI1 knockdown (Fig. S2 A-B). Western blot analysis of epithelial mesenchymal transformation (EMT) related proteins revealed increased N-cadherin, Vimentin, and Snail expression, along with decreased E-cadherin, in PC cells overexpressing PELI1. Conversely, the opposite protein expression pattern emerged after PELI1 knockdown (Fig. S2C).

### PELI1 influence on PC cell behaviour in vivo

**Fig. 3:**
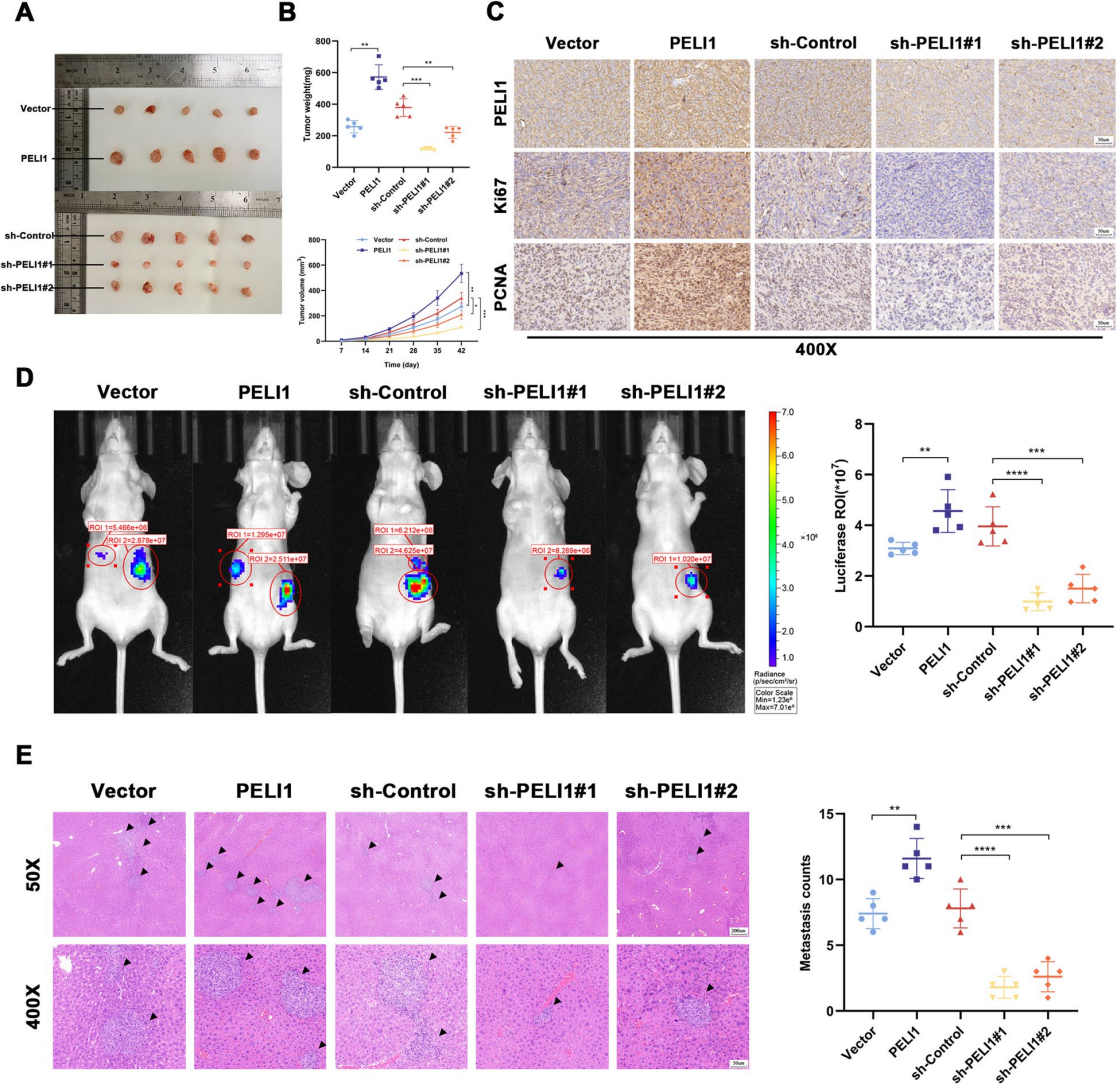
PELI1 Promotes Proliferation and Metastasis of PC Cells In Vivo. **A**, **B** Tumors formed in each group through subcutaneous tumor formation assay (**A**), including body weight and weekly changes in tumor volume (**B**). **C** IHC detection of PELI1, Ki67, and PCNA expression in tumor tissues of each group. **D** Effect of PELI1 on PC cell metastasis spleen-liver metastasis model in nude mice. **E** HE staining of liver metastases in nude mice in each group. *P < 0.05, **P < 0.01, ***P < 0.001, ****P < 0.0001

To assess whether PELI1 similarly influences PC progression in vivo, subcutaneous tumorigenesis experiments in nude mice were conducted using PELI1 stably transfected PANC-1 cells. The results unveiled larger and heavier subcutaneous tumors with faster growth rates in PC cells overexpressing PELI1, while PELI1 knockout led to smaller, lighter tumors with slower growth rate (Fig. 3A-B). IHC staining of the tumors indicated higher expression levels of PELI1, Ki67, and PCNA in tumors overexpressing PELI1, contrasting with lower expression levels in tumors with PELI1 knockdown (Fig. 3C). In a further study of patients’ PC tissues, we found that the positive rates of PELI1 and Ki-67, a proliferation-related indicator, were higher in PC tissues than in the paracancerous samples, and there was a positive correlation between the expression of PELI1 and the positive rate of Ki67 in PC tissues (Fig. S2D).

The intrasplenic injection to construct a liver metastasis model enabled in vivo observations through an animal imaging system. The results demonstrated more extensive liver metastatic foci following PELI1 overexpression, while PELI1 knockdown attenuated metastatic ability (Fig. 3D). Histological examination of fixed paraffin-embedded livers revealed a higher number of metastatic foci in the liver after PELI1 overexpression and fewer metastatic foci after PELI1 knockdown (Fig. 3E).

### PELI1 interaction with RPS3 at the FHA structural domain

**Fig. 4:**
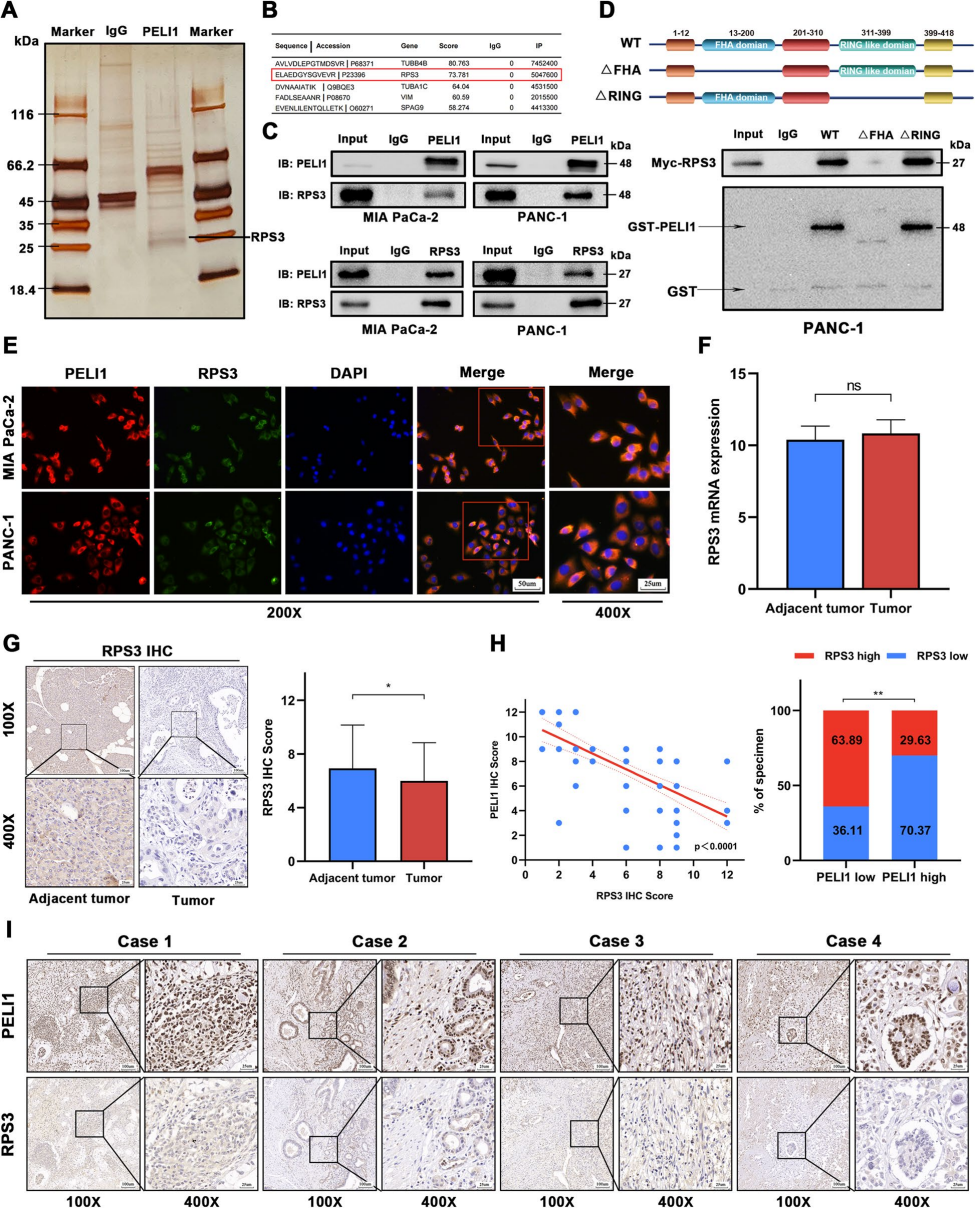
RPS3 Interaction with PELI1. **A**, **B** Proteins associated with PELI1 in PC PANC-1 cells overexpressing PELI1 by silver staining (**A**) and mass spectrometry analysis (**B**). **C** Endogenous immunoprecipitation verifying the binding of PELI1 to RPS3. **D** Schematic diagram of the deletion of different structural domains of PELI1, GST pull-down assay the inability of RPS3 to bind to PELI1 lacking the FHA structural domain. **E** Co-localization analysis of RPS3 and PELI1 in the cytoplasm using immunofluorescence. **F**, **G** qRT-PCR (**F**) and IHC (**G**) to detect the expression of RPS3 in PC and paracarcinoma tissues. **H**, **I** Immunohistochemistry showing a negative correlation between the expression of RPS3 and PELI1 protein levels. *P < 0.05, **P < 0.01

To explore potential reciprocal proteins of PELI1 in PC, we identified a potential interaction between PELI1 and RPS3 through protein profiling (Fig. 4A-B). Subsequent endogenous immunoprecipitation experiments validated the interaction between PELI1 and RPS3 (Fig. 4C). Further clarification was achieved using a GST pull-down assay, revealing that PELI1 lacking the FHA domain failed to interact with RPS3 (Fig. 4D).

Deletion of the FHA structural domain was found to diminish PELI1’s regulatory impact on the proliferative capacity of PC cells, as demonstrated by CCK8, plate cloning, and EdU experiments(Fig. S3A-C). Immunofluorescence co-localization confirmed predominant cytoplasmic localisation of PELI1 and RPS3 (Fig. 4E). qRT-PCR tests indicated no significant alterations in RPS3 transcript levels in PC cell lines overexpressing or knocking down PELI1 (Fig. S3D), meanwhile RPS3 mRNA transcripts were not significantly different in PC and matching paracancerous tissues (Fig. 4F). But IHC results revealed lower protein expression of RPS3 in PC tissues compared to paracancerous tissues (Fig. 4G). IHC co-localization staining demonstrated a negative correlation between the IHC staining of PELI1 and RPS3 in PC tissues (Fig. 4H-I), suggesting that RPS3 might undergo post-translational modification by PELI1.

### PELI1 control of RPS3 protein stability

**Fig. 5:**
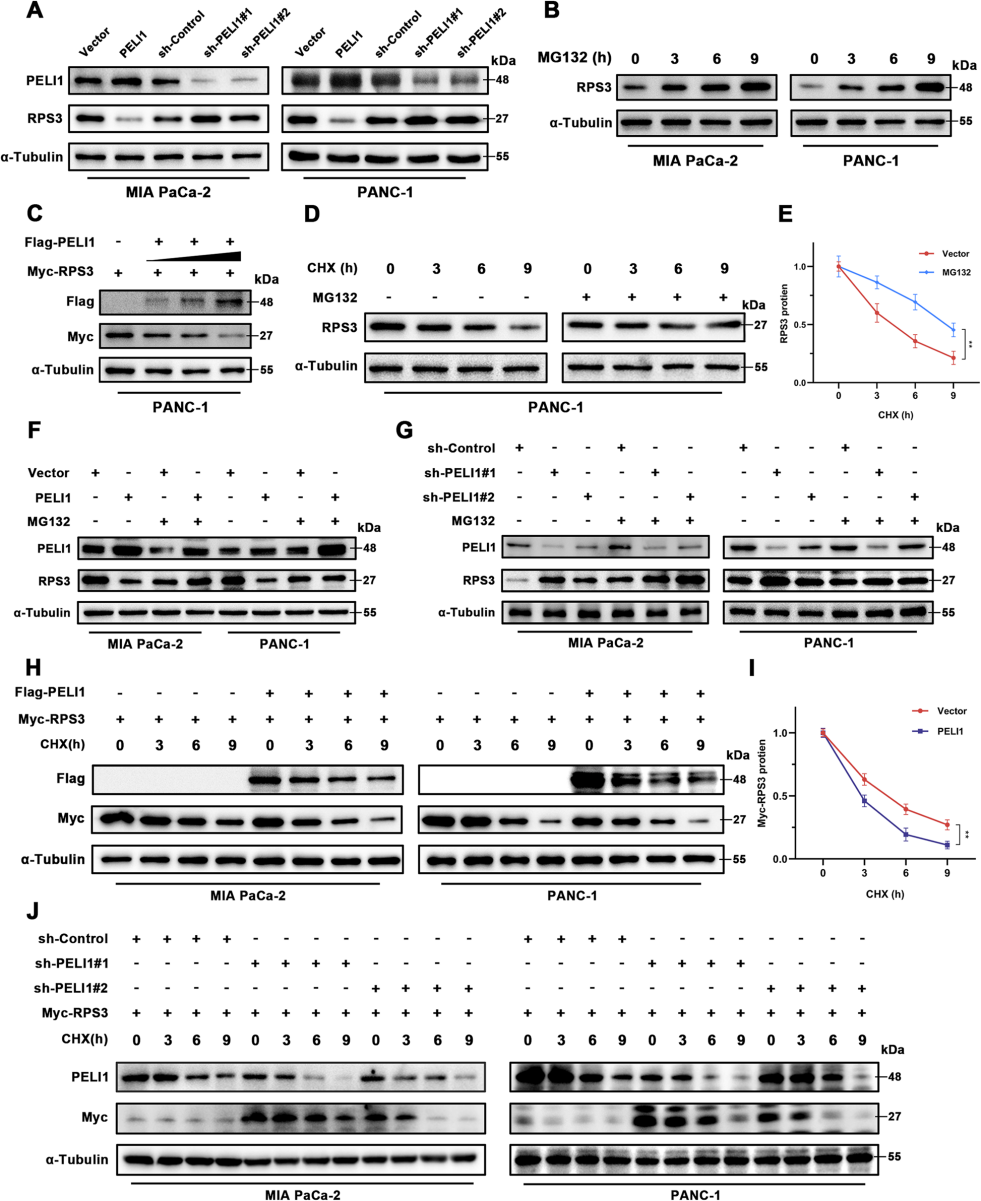
PELI1 Promotes RPS3 Degradation. **A** Western blot assay for the relationship between PELI1 and RPS3 expression in PC cells. **B** Increased RPS3 expression over time with increasing MG132 (20 μM) action time. **C** Decreased expression of exogenous RPS3 with increasing transfection of the Flag-PELI1 plasmid. **D**, **E** Prolongation of RPS3 protein half-life after MG132 (20 μM) treatment. **F**, **G** The degradation of RPS3 protein by PELI1 was inhibited after MG132 (20 μM) treatment. **H**, **I** PC cells transfected with Flag-PELI1, Myc-RPS3, and treated with CHX (20 μM) show increased RPS3 degradation in the PELI1 group. **J** RPS3 degradation slows down in the knockdown PELI1 group after CHX (20 μM) treatment. **P < 0.01

To assess the influence of E3 ubiquitin ligase PELI1 on RPS3 protein stability, Western blot analysis was employed to measure RPS3 protein expression in PC cells after PELI1 overexpression or knockdown. Results indicated a decrease in RPS3 protein expression with PELI1 overexpression and an increase with PELI1 knockdown (Fig. 5A). Protein stability studies revealed a time-dependent rise in RPS3 protein expression in response to the proteasome inhibitor MG132 (Fig. 5B), indicating RPS3 protein degradation primarily via the ubiquitin-proteasome pathway.

Further transfection of exogenous plasmid was demonstrated a decrease in exogenous RPS3 protein expression with increasing amounts of PELI1 plasmid (Fig. 5C). The half-life of RPS3 protein was extended following MG132 treatment in PANC-1 cells (Fig. 5D-E). Interestingly, no significant change was observed in RPS3 expression in PC cells treated with MG132, regardless of PELI1 overexpression or knockdown (Fig. 5F-G). Overexpression of PELI1 resulted in a reduction in the half-life of exogenous RPS3 protein in PC cell lines treated with CHX, whereas PELI1 down-regulation extended the protein half-life of exogenous RPS3 (Fig. 5H-J). These findings collectively support the notion of PELI1-mediated degradation of RPS3.

**Fig. 6:**
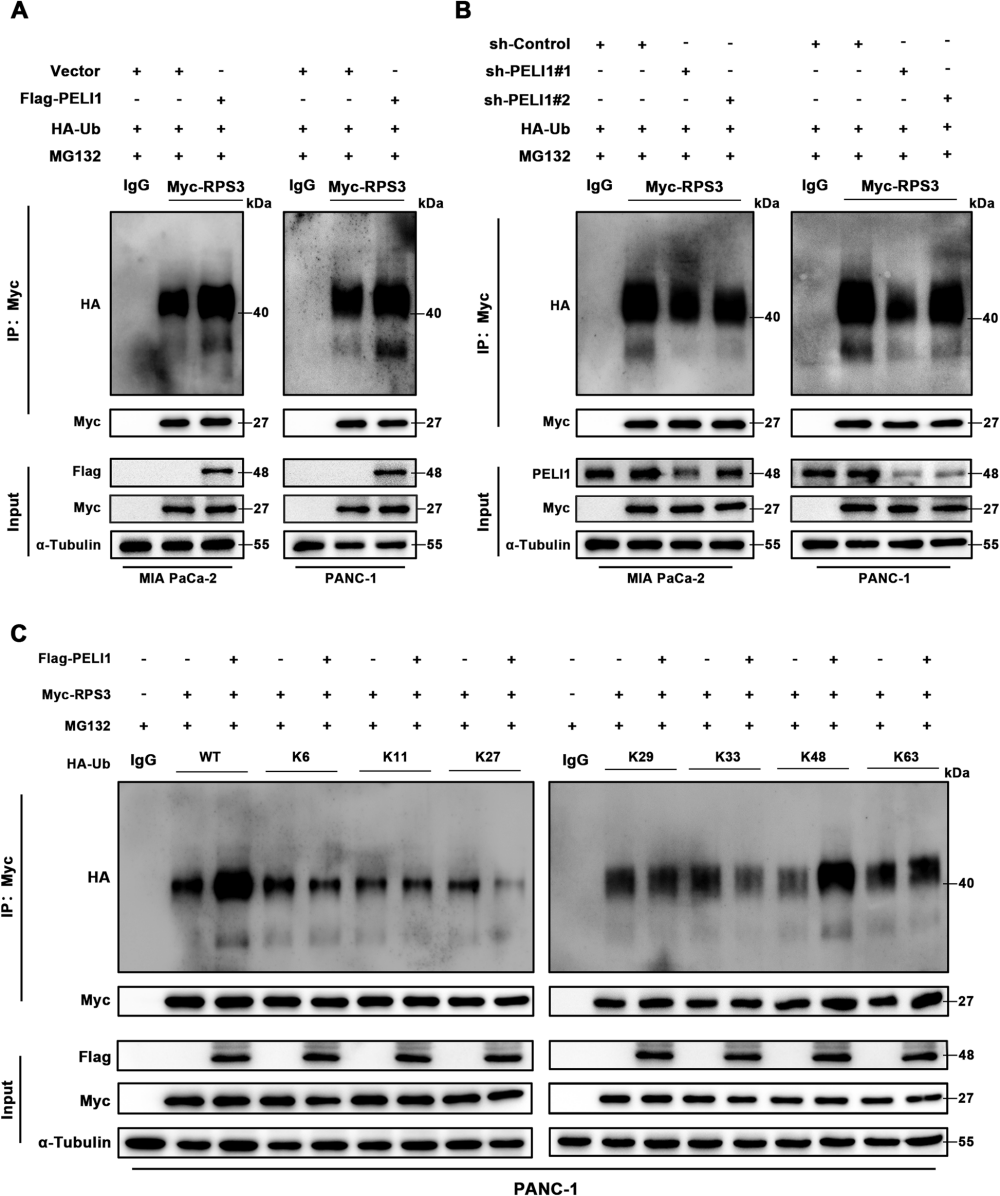
PELI1 Promotes Polyubiquitination of RPS3 in the K48 Chain. **A**,** B** PC cells transfected with HA-Ub, with or without Flag-PELI1 plasmid, and treated with MG132 (20 μM), demonstrating the level to ubiquitination of RPS3. **C** Detection of PELI1 polyubiquitinates RPS3 at the K48 chain, using Flag-PELI1, Myc-RPS3, HA-WT/K6/K11/K27/K29/K33/K48/K63

To demonstrate the impact of PELI1 on RPS3 ubiquitination, a ubiquitination experiment was conducted. The results indicated elevated ubiquitination of RPS3 in PELI1 overexpresion PC cells and reduced ubiquitination level in PELI1 knockdown PC cells (Fig. 6A-B). Transfection of HA-Ub plasmids with different ubiquitin chains in PC cells suggested that PELI1 facilitated RPS3’s polyubiquitination on the K48 chain (Fig. 6C).

### PELI1 regulation of PC cell malignancy via the PI3K-Akt-GSK3β signaling pathway

**Fig. 7:**
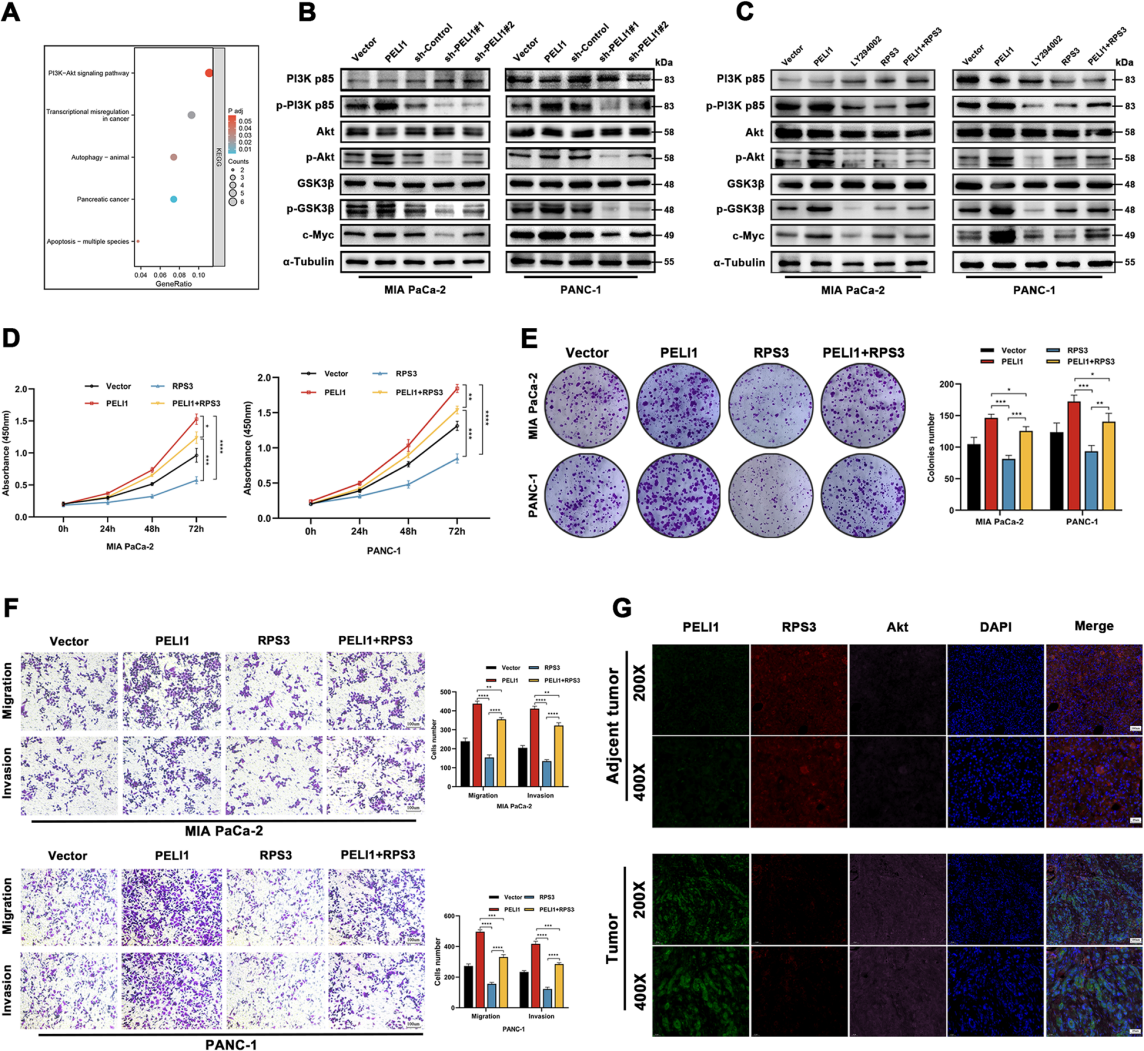
PELI1 Promotes PI3K/Akt/GSK3β Pathway Activation through RPS3. **A** KEGG enrichment analysis indicates the potential association of PELI1 with the PI3K/Akt pathway. **B** PELI1 promotes the phosphorylation of key proteins in the PI3K/Akt/GSK3β pathway. **C** Treatment with the PI3K inhibitor LY294002 and overexpression of RPS3 lead to a reduction in the phosphorylation of key proteins in the PI3K/Akt/GSK3β pathway. **D**, **E** The effects of RPS3 on PC cell proliferation assessed by CCK-8 (D), clone formation (E). **F** Effects of RPS3 on PC cell metastasis and invasion were examined by Transwell assay. **G** Multiplex fluorescence IHC assesses protein expression of PELI1, RPS3, and Akt in PC and paracancerous tissues. *P < 0.05, **P < 0.01, ***P < 0.001, ****P < 0.0001

To elucidate the pro-carcinogenic mechanism of PELI1 in PC, a KEGG enrichment analysis was performed on PAAD transcripts from the TCGA database, revealing significant activation of the PI3K-Akt signaling pathway by PELI1 (Fig. 7A). Western blot validation demonstrated increased protein expression levels of p-PI3K (p85), p-Akt, p-GSK3β, and c-Myc with PELI1 overexpression and decreased expression with PELI1 knockdown (Fig. 7B). Subsequent rescue tests indicated that PI3K inhibitors LY292004 and RPS3 had inhibitory effects on the expression of p-PI3K (p85), p-Akt, p-GSK3β, and c-Myc proteins (Fig. 7C). These results suggest that PELI1 may ubiquitinate RPS3, consequently activating the PI3K-Akt-GSK3β pathway and promoting the malignant behavior of PC cells.

CCK8, clone formation, and EdU assays collectively demonstrated that upregulation of RPS3 inhibited the proliferative capacity of PC cells (Fig. 7D-E, Fig. S4B). Further investigations revealed that overexpression of RPS3 in PC cells led to decreased expression of Cyclin D1, Cyclin E1, CDK7, and CDK4, along with increased expression of p27 (Fig. S4A). Moreover, wound healing and transwell assays indicated that increased RPS3 expression decreased the metastatic and invasive potential of PC cells (Fig. S4C, Fig. 7F). Multiple fluorescent IHC staining showed relatively high expression of PELI1 and Akt and relatively low expression of RPS3 in PC tissues and opposite expression of the above proteins in matched paracarcinoma tissues (Fig. 7G). Subcutaneous tumors in nude mice overexpressing PELI1 showed increased protein expression of PELI1 and Akt and decreased expression of RPS3 (Fig. S5A). Additionally, the introduction of the PI3K inhibitor LY294002 to PC cells resulted in inhibited proliferative ability (Fig. S5B-D). The findings suggested that PELI1 plays a role in enhancing PC proliferation, invasion, and metastasis by regulating RPS3 ubiquitination and thereby activating the PI3K-AKT-GSK3β pathway.

### PELI1-mediated p53 degradation via RPS3 regulation

**Fig. 8:**
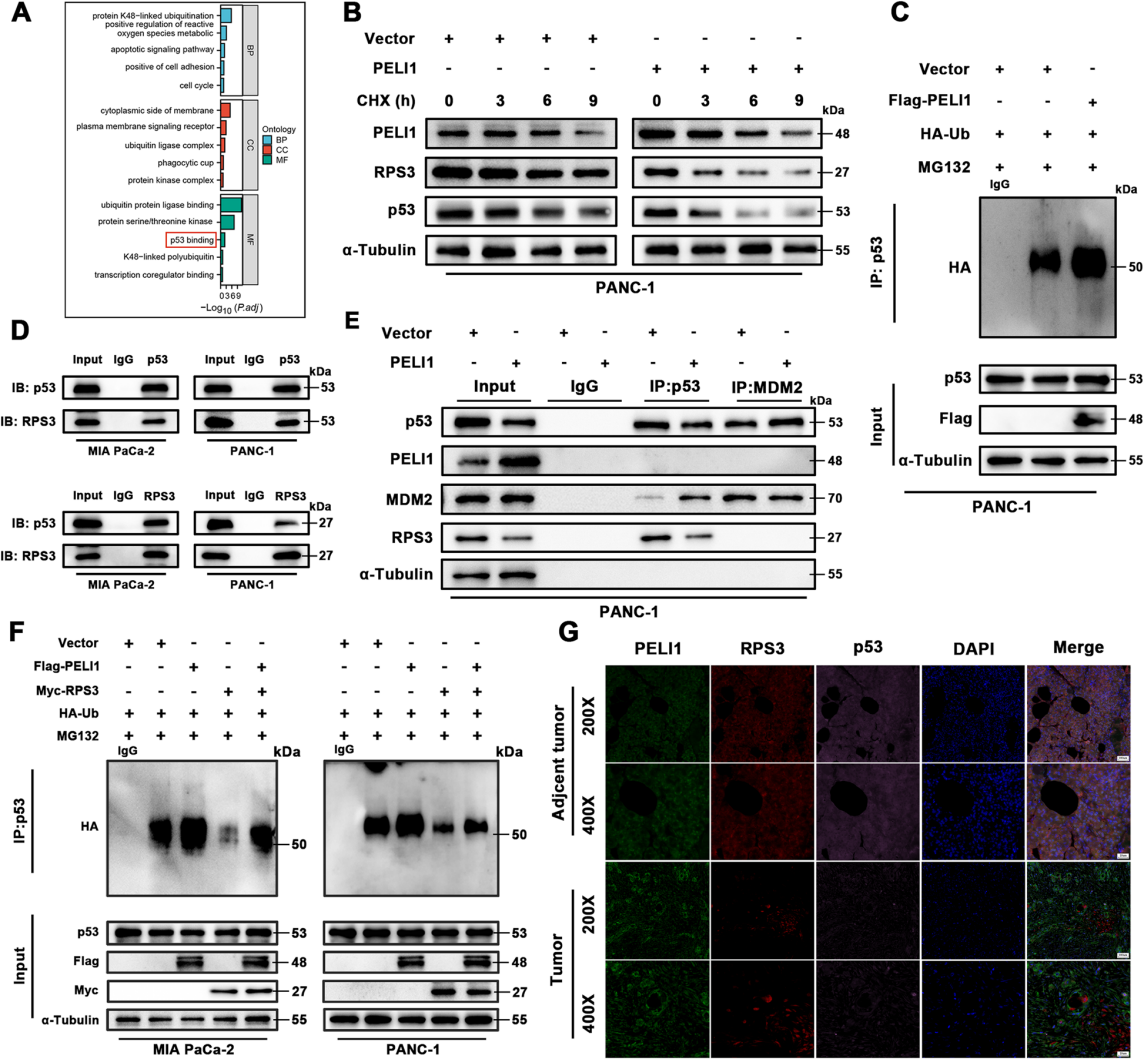
PELI1 Promotes p53 Degradation through RPS3. **A** GO enrichment analysis reveals an association between RPS3 and p53 binding. **B** Increased rate of p53 and RPS3 degradation in the PELI1 overexpression group after CHX (20 μM) treatment. **C** PC cells transfected with HA-Ub, with or without Flag-PELI1 plasmid, show elevated ubiquitination of p53 after MG132 (20 μM) treatment. **D** Endogenous immunoprecipitation confirms the binding of RPS3 to p53. **E** Immunoprecipitation with or without Flag-PELI1 plasmid, utilizing p53 and MDM2 antibodies, indicates that PELI1 decreases the binding of p53 to RPS3, while increasing p53 binding to MDM2. **F** PC cells transfected with HA-Ub, with or without Flag-PELI1 and Myc-RPS3 plasmids, and treated with MG132 (20 μM), demonstrate reduced ubiquitination of p53 with RPS3 overexpression. **G** Multiplex fluorescent IHC evaluates the protein expression of PELI1, RPS3, and p53 in PC and paracancerous tissues

In further exploration of PELI1 and RPS3 regulatory roles in PC, an additional potential oncogenic mechanism was identified. GO enrichment analysis indicated that RPS3’s molecular function (MF) may involve binding to p53 (Fig. 8A). Western blot assays demonstrated that PELI1 overexpression significantly reduced p53 protein expression levels (Fig. 8B), suggesting that PELI1 may influence p53 stability. Ubiquitination experiments further revealed that PELI1 overexpression increased the ubiquitination level of p53 (Fig. 8C).

Given that p53 ubiquitination is regulated by MDM2, thus influencing various cellular processes and pathways [30], we hypothesized that PELI1 might affect p53 ubiquitination through RPS3-mediated MDM2. Co-IP experiments confirmed the endogenous interaction between RPS3 and p53 (Fig. 8D). Upon PELI1 overexpression, the binding ability of RPS3 to p53 weakened, while the binding ability of MDM2 to p53 strengthened. However, there was no direct interaction between PELI1, MDM2, p53, and between MDM2 and RPS3 (Fig. 8E). Overexpression of RPS3 significantly reduced the ubiquitination level of p53 (Fig. 8F). In addition, further studies revealed that overexpression of PELI1 promoted polyubiquitination of p53 on the k48 chain, while decreasing polyubiquitination on the K63 chain, whereas there was no significant change in the protein level of MDM2 (Fig. S6A). These results suggest that PELI1 may regulate the binding of RPS3 to p53, thereby affecting the degradation of p53 ubiquitination by MDM2.

Multiplex fluorescence IHC staining indicated decreased levels of RPS3 and p53 proteins in PC tissues compared to corresponding paracancerous tissues (Fig. 8G). Subcutaneous tumors overexpressing PELI1 exhibited lower levels of RPS3 and p53 protein expression compared to the blank control (Fig. S6B).

## Discussion

**Fig. 9:**
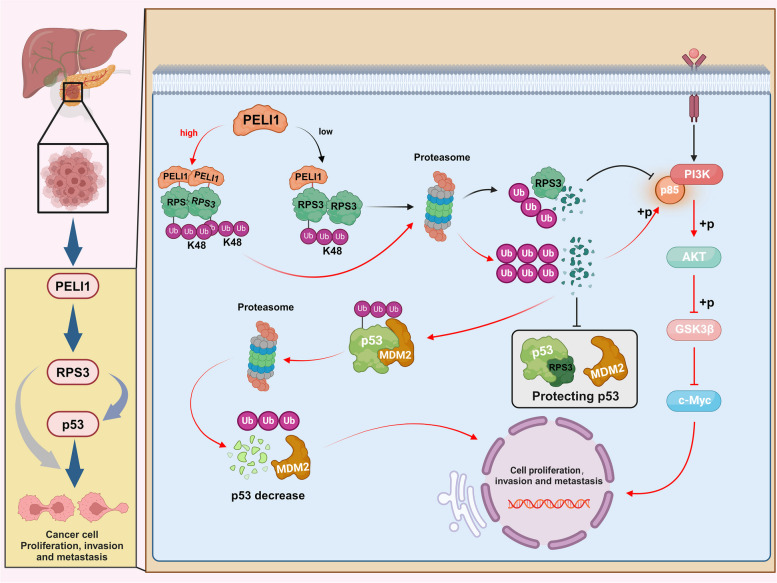
Mechanistic map of PELI1 ubiquitination of RPS3 activating the PI3K/AKT/GSK3β signaling pathway, promoting p53 degradation, and thus facilitating progression in PC. Created by BioRender. (Agreement number: OZ26EQJDYS)

PC stands as the most malignant neoplasm within the spectrum of digestive tract tumors, characterized by a low rate of successful one-stage surgical resection and a marked tendency for recurrence. Adjuvant therapy, though now an integral part of the treatment regimen for the majority of PC patients, demonstrates limited efficacy in terms of patient outcomes and survival [31]. Investigating the intricate mechanisms underpinning PC and identifying targets capable of effectively impeding its progression are thus of paramount importance. Currently, the utilisation of ubiquitin proteasome inhibition in tumour therapy has shown promising outcomes [32, 33]. A comprehensive exploration of the pro-cancer mechanisms of the UPS in PC could provide valuable insights for molecular interventions in PC treatment Fig. 9.

PELI1, an E3 ubiquitin ligase, has been implicated in the pro-cancer progression of various malignant tumours. This study delves into the role of PELI1 in activating the PI3K/AKT/GSK3β pathway through the ubiquitination modification of RPS3, concurrently participating in the degradation of p53 to propel the malignant behaviour of PC. Initial scrutiny involved bioinformatics analysis of public databases, integrating clinical data and tissue specimens from PC patients, and analysis of PELI1 expression levels in different cell lines. The findings revealed PELI1’s overexpression in both PC tissues and cell lines, correlating positively with adverse patient prognosis. Subsequent in vivo and in vitro experiments substantiated that elevated PELI1 expression promotes the proliferation and metastasis of PC cells. Notably, the FHA structural domain of PELI1 was identified to bind to and ubiquitinate RPS3, a potential target, thus influencing its degradation and activating the PI3K-Akt-GSK3β pathway. Concurrently, PELI1’s ubiquitination of RPS3 enhances MDM2 binding to p53, instigating p53 protein degradation and facilitating PC progression.

PELI1 is known to play an important role in inflammation and autoimmunity [34],and also been found to play a pro-cancer role in malignant tumors. In oesophageal cancer, PELI1 has been shown to mediate K48 chain polyubiquitination, inhibiting the atypical NF-κB pathway and modulating tumour cell sensitivity to radiotherapy [24]. Furthermore, PELI1 maintains cellular stability by inhibiting aberrant cell death induced by RIP3 overexpression through mediated polyubiquitination of RIP3 on the K48 chain [35].

Our findings underscore the close association of PELI1 high expression in PC with tumour size and pathological stage. Cox risk-proportional regression modelling identified PELI1 as an independent risk factor impacting the prognosis of PC, suggesting a pivotal role for PELI1 in PC progression. Subsequent in vivo and in vitro experiments with PC cell lines confirmed that PELI1 overexpression enhances the proliferation and metastasis of PC cells, aligning with existing literature that positions PELI1 as an oncogene in solid tumours. These results fortify the hypothesis that PELI1 functions as an oncogene in PC, presenting it as a potential novel therapeutic target. In addition, PELI1 is also involved in T cell metabolism and anti-tumor immunity, and regulates T cell infiltration [36].These roles of PELI1 may also contribute to the poor results of immunotherapy for PC. At present, we haven’t studied in depth in connection between PELI1 and PC tumor immunity-related cells, but this will be our next step in our work plan.

Our investigation uncovered a post-translational modification effect of PELI1 on RPS3, a multifunctional ribosomal protein pivotal in cellular homeostasis. Previous research has indicated that increased intracellular ROS leads to mitochondrial RPS3 aggregation, facilitating the repair of damaged mitochondrial DNA [37]. Additionally, ubiquitination of RPS3 has been linked to enhanced resistance of glioblastoma cells to radiotherapy and decreased apoptosis [29]. Our research revealed predominant cytoplasmic localisation of RPS3 in PC, with comparatively lower protein expression in PC than in adjacent normal tissues. PELI1, binding to RPS3 through the FHA structural domain, mediated increased expression of phosphorylated proteins associated with the PI3K/Akt/GSK3β signalling pathway through polyubiquitination of the K48 chain. This pathway plays a crucial role in the development of various human diseases, including malignant tumours [38, 39]. GSK3, an influential factor in tumor cells, impacts multiple biological processes such as metabolism and cell proliferation regulation [40]. Rescue experiments underscored the role of RPS3 in PC, revealing that its overexpression inhibited the PI3K-Akt-GSK3β pathway, subsequently impeding the proliferation and metastatic ability of PC cells in vitro.

Furthermore, our findings suggest a correlation between RPS3 and the maintenance of p53 stability. Notably, earlier research underscores the crucial role of ribosomal proteins in balancing the MDM2-p53 axis and promoting apoptosis through p53 or related pathways [41, 42]. The absence or abnormal function of p53 can promotes the progression of PC [43], and p53 abnormal is present in most PC [44]. In our study, a decrease in RPS3 protein level correlated with an increase in p53 ubiquitination, indicating an interaction between RPS3 and p53. Rescue experiments demonstrated that RPS3 overexpression reduced p53 ubiquitination, suggesting aa protective role on the p53 protein. We hypothesized that binding of RPS3 to p53 involves competitively inhibiting the binding rate of p53 to MDM2, thereby protecting and reducing MDM2-mediated p53 degradation. Co-IP experiments showed that PELI1 overexpression decreased RPS3-p53 binding while increasing p53-MDM2 binding, supporting our hypothesis and revealing an extra-ribosomal role for RPS3 in stabilizing the p53 protein.

In conclusion, our study highlights the crucial role of PELI1 in PC, identifying its overexpression and association with shortened survival in PC patients. Both in vivo and in vitro experiments underscored PELI1’s function as an oncogene in PC. We unraveled the regulatory mechanism of PELI1-RPS3 ubiquitination, elucidating its impact on the activation of the PI3K/Akt/GSK3β pathway and increased degradation of the p53 protein, thereby promoting PC cell proliferation and metastasis. These findings suggest PELI1 as a potential therapeutic target for PC, holding promise for enhanced patient benefits through targeted interventions.

## Material and methods

### Bioinformatics analysis

Gene expression transcripts (RNA-seq) and clinical data from 179 PAAD and 4 paracancerous tissues were sourced from TCGA (https://docs.gdc.cancer.gov/), while 167 normal pancreatic gene transcripts were obtained from the GTEx database (https://gtexportal.org/home/). Additionally, microarray expression data from the GEO database (https://www.ncbi.nlm.nih.gov/geo/) included GSE62165, GSE15471, and GSE71989 datasets. Transcript differential analysis employed “limma,” “edgeR,” and “stats” for statistical analysis, and data visualization utilized the “ggplot2” software package. Survival analysis employed the “survival” package, with survival curves plotted using “survminer” and “ggplot2.” ROC analysis was conducted using the “pROC” package, and results were visualized using “ggplot2.”

### Specimen collection

Ninety PC tissue and paracancerous specimens (1–2 cm of normal pancreatic tissue adjacent to tumor parenchyma) with clinicopathologic data were procured from patients at the Hepatobiliary Surgery Department of the Affiliated Hospital of Guizhou Medical University and the Affiliated Tumor Hospital, who underwent surgical resection from April 1, 2020, to April 1, 2023. Ethical approval for specimen collection and clinical data was granted by the Ethics Committee of Guizhou Medical University (2019 Ethical Review No. 073), and participating patients provided informed consent orally and through written agreements.

### Cell culture

The normal human pancreatic ductal epithelial cells hTERT-HPNE and PC cell lines (AsPC-1, BxPC-3, PANC-1, MIA PaCa-2, SW1990) were procured from the Cell Bank of the Chinese Academy of Sciences. Culturing conditions included DMEM (Gibco, USA) with 10% FBS (Gemini, USA) and 1% penicillin for hTERT-HPNE, PANC-1, MIA PaCa-2, and SW1990, while AsPC-1 and BxPC-3 were cultured in RPMI 1640 (Gibco, USA) with 10% FBS and 1% penicillin. All cells were maintained in a 37 °C, 5% CO_2_, 95% air incubator (Thermo, USA).

### Stabilized cell line and plasmid construction and transfection

Lentiviruses including negative control lentivirus Vector (Lv-NC), PELI1 overexpression lentivirus (Lv-PELI1), PELI1 knockdown lentiviruses (sh-NC, sh-PELI1#1, sh-PELI1#2), and Myc-RPS3 were obtained from Shanghai Genechem Co. Ubiquitin (HA-Ub) overexpression plasmids (HA-WT, HA-K6, K11, K27, K29, K33, K48, K63) were purchased from Shanghai Sangon Bioengineering Co. Plasmid transfections utilized lipofectamine 3000 (Invitrogen, USA) and Opti-MEM (Gibco, USA).

### Antibodies and reagents

During the study, various antibodies were employed, including PELI1 (ab199336, Abcam) for Western blot (WB), immunohistochemistry (IHC), immunofluorescence (IF), and immunoprecipitation (IP), Cyclin E1 (11554–1-AP, Proteintech), Cyclin D1 (60186–1-Ig, Proteintech), CDK7 (#2916, CST), CDK4 (GB111602–100, Servicebio), p27 (25614–1-AP, Proteintech), E-cadherin (20874–1-AP, Proteintech), N-cadherin (22018–1-AP, Proteintech), Vimentin (10366–1-AP, Proteintech), Snail (10399–1-AP, Proteintech), Alpha Tubulin (66031–1-Ig, Proteintech), RPS3 (66046–1-Ig for WB/IHC/IF, 11990–1-AP for IP, Proteintech), Ki67 (27309–1-AP, Proteintech), PCNA (10205–2-AP, Proteintech), p53 (66031–1-Ig for WB, 10442–1-AP for IP/mIHC, Proteintech), Flag (66008–4-Ig, Proteintech), HA (66006–2-Ig, Proteintech), Myc (60003–2-Ig, Proteintech). Fluorescent secondary antibodies included mouse secondary antibody (SA00009–1, Proteintech) and rabbit secondary antibody (SA00013–4, Proteintech).

Chemical reagents employed in the experiment consisted of CHX (Aladdin, China), MG132 (Sigma, USA), four-color multiplex immunohistochemistry kit (Absin, abs50012, China), PI (Solarbio, China), DAPI (Solarbio, China), and D-Luciferin Potassium salt (PerkinElmer, USA).

### Immunohistochemistry and multiple fluorescence immunohistochemistry

Tissues were fixed, dehydrated, and paraffin-embedded with 4 μm thick sections. Sections were deparaffinized with xylene, hydrated with an ethanol gradient, and subjected to antigen repair using EDTA (Solarbio, China). Endogenous peroxidase was blocked with H_2_O_2_, followed by confinement with 5% BSA (Solarbio, China). Antibodies were applied overnight at 4 °C, followed by washing with PBST and incubation with secondary antibodies at room temperature for 2 hours. Sections were developed with diaminobenzidine working solution (ZSGB-Bio, China), dehydrated with an ethanol gradient, and transparently sectioned using xylene. Slides were sealed with neutral resin (Solarbio, China). IHC scoring was performed by two pathologists based on staining intensity and cell count.The intensity of cell staining was scored on a 4-point scale, (negative = 0, weakly positive = 1, positive = 2, strongly positive = 3), and the percentage of positive cells was scored on a 5-point scale, < 1% = 0, 1–25% = 1, 26–50% = 2, 51–75% = 3, and > 75% = 4. The two scores were then multiplied to give a final score. Scores below less than 7 were considered low expression, and scores greater than or equal to 7 were considered high expression.

For multiple fluorescence IHC, the same treatment as IHC staining was applied, with an additional incubation step using staining solution. After antibody staining, DAPI was applied for nuclei staining, and slides were sealed with an anti-fluorescence quencher. TBST washes were performed between each step, and fluorescence intensity was observed under a laser confocal microscope (Olympus, Japan).

### Hematoxylin-eosin (HE) staining

Livers from mice in intrasplenic injection to construct a liver metastasis model were fixed, dehydrated, paraffin-embedded, and sectioned. Deparaffinization, hydration, and staining with hematoxylin and eosin were performed, followed by dehydration, xylene transparency, and blocking with neutral resin. Metastatic foci were counted through photography under an inverted microscope (Nikon, Japan).

### RNA extraction and qRT-PCR experiments

TRIzol (Invitrogen, USA) reagent was used to extract total RNA from PC tissues and cells, and a NanoDrop spectrophotometer (Thermo, USA) was used to measure the concentration and mass of RNA with A260/A280 in the range of 1.8–2.0. RNA was reverse transcribed to cDNA using a PrimeScript RT kit (TaKaRa, Japan), and mRNA expression was analyzed using a Premix Ex TaqTM (Takara, Japan) kit and a real-time fluorescence quantitative PCR instrument. The Forward primer for PELI1 (5′- GAAATTGGTCCCCAGCCCTC-3′), Reverse primer (5′-GCGAGAGGGTGAAGTGTTGT-3′), Forward primer for α-Tubulin (5 ‘-ACCAACCTGGTGCCCTATCC-3’), Reverse primer (5′-CAAGCATTGGTGATCTCTCTGCTACA-3′). The Forward primer for RPS3 (5′-TACTACGTTGACACTGCTGTGCG-3′), Reverse primer (5′-GGTGGTGGGCAGTATCTCATCTT-3 ‘).

### Western blot assay

Total protein was extracted from cells or tissues by adding a mixture of RIPA lysis buffer (Solarbio, China) and protease inhibitor (Boster, China) at 4 °C with centrifugation at 12,000 rpm for 15 min. The supernatant was collected, and protein quantification was performed using the BCA kit (Solarbio, China). Proteins were denatured at 95 °C, followed by sodium dodecyl sulfate-polyacrylamide gel electrophoresis (SDS-PAGE) and transfer to PVDF membranes (Meck, USA). After blocking with 5% skimmed milk powder at room temperature for 2 h, overnight incubation with primary antibodies at 4 °C, and subsequent incubation with secondary antibodies at room temperature for 2 h, membranes were washed with TBST containing 0.1% Tween (Solarbio, China). ECL chemiluminescent solution (Millipore, USA) was applied, and protein expression levels were analysed using the ChemiDocTM imager (Bio-Rad, USA).

## Cell proliferation assay - CCK8 assay

PC cell density was adjusted and cells were inoculated into 96-well plates (3000 cells/well). After 0, 24, 48, and 72 h, CCK8 reagent (GlpBio, USA) was added, incubated for 2 h at 37 °C, and absorbance was measured at 450 nm using an enzyme marker (Thermo, USA).

### EdU proliferation assay

PC cells (3 × 10^4^ cells/well) were inoculated in 24-well plates. When cells reached 70–80% density, EdU storage solution (20 μM, Servicebio, China) was added and incubated for 2 h. Cells were fixed with 4% paraformaldehyde (Solarbio, China), permeabilised with 0.5% Triton-X (Solarbio, China), stained with IF 555 for EdU, and nuclei were stained with DAPI. Images were captured using a fluorescence microscope (Nikon, Japan).

### Clone formation experiment

PC cells (1000 cells/well) were inoculated in 6-well plates, cultured for 14 days, fixed with 4% paraformaldehyde solution, stained with 0.3% crystal violet, washed, dried, and photographed for counting.

### Cell cycle detection by flow cytometry

PC cells were inoculated in 6-well plates, digested, centrifuged, fixed with pre-cooled 70% ethanol at − 20 °C overnight, stained with RNase A and PI, and incubated for 30 minutes before detection by flow cytometry (Beckman, USA) according to the Cell Cycle Instruction (CA1510, Solarbio, China).

### Wound healing experiment

PC cells were cultured to approximately 100% density in 6-well plates, scratched uniformly using a 200 μl sterile tip, rinsed with PBS, and incubated in serum-free medium. Alterations in cell wounding were observed at 0 and 48 hours post-scratch under an inverted microscope.

### Transwell experiments

PC cells were starved with serum-free medium for 12 h, inoculated in the upper chamber, and 20% FBS DMEM medium was added to the lower chamber. After incubation at 37 °C for 24–28 hours, cells were fixed, stained with crystal violet, and counted by inverted microscope observation. For invasion experiments, 60 μl of Matrigel was added to the upper chamber and left at 4 °C overnight.

### Immunofluorescence assay

Cells in the logarithmic growth phase were carefully chosen and cultured on slides for 8 h. Fixation was achieved with 4% paraformaldehyde, followed by a 20 min incubation with 0.5% TritonX-100 at room temperature. Slides were sealed with 5% BSA for 30 min, and the corresponding primary antibody was added, incubating in a wet box at 4 °C overnight. After discarding the liquid, the fluorescence secondary antibody specific to the species was applied and incubated at room temperature, shielded from light, for 2 h. DAPI staining for 5 min and sealing with an anti-fluorescence quencher concluded the process. The samples were washed three times with PBS after each step and photographed under a fluorescence-inverted microscope.

### Immunoprecipitation and ubiquitination experiments

NP40 lysate, combined with protease inhibitor, phosphatase inhibitor, and EDTA, was added to PC cells for detection. Lysis was performed on ice for 30 min, followed by centrifugation at 12,000 rpm for 10 min to collect the clarified protein solution. Protein A + G (beyotime, China) was added for pre-purification for 4 hours at 4 °C on a shaking bed at 2500 rpm. The upper layer of the protein solution was extracted, and the corresponding antibody, along with 20–40 μl of protein A + G, was incubated at 4 °C on a shaking bed overnight. After centrifugation of the protein, antibody and magnetic bead complex layer (4 °C, 2500 rpm, 5 min), washing with PBS was performed three times. To denature the protein-antibody complex and detach it from the magnetic beads, a 95 °C treatment for 10 min was employed. Finally, the obtained samples underwent Western blot analysis.

### Ubiquitination assay

Cells were cultured in 6-well plates, and when the cell density reached 70–80%, transfection with the corresponding HA-Ub, Myc-RPS3 overexpression, and Flag-PELI1 overexpression plasmids was conducted. The solution was changed 8 hrs post-transfection, and the next day, 20 μM MG132 was added. After 8 h of cultivation, cells were digested by 0.25% trypsin, and the protein-antibody complexes were extracted using the Co-IP method. Finally, the ubiquitination level of the corresponding proteins was detected using Western blot.

### Subcutaneous tumor formation in nude mice

Twenty-five 5–6 week-year female nude mice (BALB/c-Nude, Gempharmatech, China) were meticulously chosen and randomly assigned into five groups (Vector, PELI1, sh-Control, sh-PELI1#1, sh-PELI1#2). Subsequently, 2 × 10^6^ PANC-1 cells, stably transfected with PELI1, were subcutaneously inoculated into the right lower limb of the corresponding subgroup of mice. Tumour size was measured weekly, and after 6 weeks of continuous rearing, the mice were euthanized with an overdose of isoflurane anesthesia. The excised tumours were utilised for subsequent IHC analysis. All animal experiments were ethically approved by the Animal Ethics Committee of Guizhou Medical University (NO. 19000672).

### Intrasplenic injection to construct a liver metastasis model in nude mice

Twenty-five 5–6 week-old female nude mice were selected and randomly distributed into five groups (Vector, PELI1, sh-Control, sh-PELI1#1, sh-PELI1#2). The nude mice were anaesthetized using isoflurane inhalation and then secured in right lateral recumbency. The skin beneath the left costal margin was disinfected, layers were clipped incrementally up to the peritoneum, and the tail of the spleen was exposed and injected with 1 × 10^6^ PC cells stably transfected with PELI1. Subsequently, the abdominal cavity was closed layer by layer. After intraperitoneal injection of Luciferase (10 μl/10 g) every 7 days for 5 min, recordings were captured using an animal live imager (PerkinElmer, USA). After 5 weeks, mice were euthanized with an isoflurane overdose, and the livers were excised and fixed for subsequent HE staining analysis.

### Statistical analyses

Statistical analyses were conducted using SPSS (version 25.0, IBM, USA) and GraphPad Prism (version 8.03, GraphPad, USA). The correlation between PELI1 expression and clinicopathological characteristics was assessed using the chi-square test. For skewed distribution data, the median (interquartile spacing) was employed. Student’s t-test (two-tailed) was utilised for comparisons between two groups, while analysis of variance (ANOVA) was applied for comparisons between multiple groups. The Kaplan-Meier method was employed for survival analysis, with the Log-rank test used to analyze between-group differences. Cox’s risk-proportionality model was implemented for multifactorial analysis, exploring the relationship between PELI1 expression and the survival time of patients with PC. A significance level of *P* <  0.05 was considered statistically different.

### Supplementary Information

**Additional file 1: Fig. S1.** Bioinformatics Analysis of the Relationship Between PELI1 Expression in PC Tissues and Prognosis. A. Analysis of PELI1 mRNA expression in PC tissues based on the GTEx and TCGA databases. B. Analysis of PELI1 mRNA expression in PC tissues using three microarray expression datasets (GSE62165, GSE15471, and GSE71989) from the GEO database. C. Association of the high expression of PELI1 with poor prognosis in PC based on the TCGA database. D. ROC analysis demonstrating PELI1's predictive value for the clinical outcome of PC patients. ***P < 0.001, ****P < 0.0001

**Additional file 2: Fig. S2.** PELI1 promotes human PC proliferation, and in vitro invasive and metastatic. A, B Wound healing assay (A) and Transwell assay (B) to detect the effects of PELI1 on PC cell metastasis and invasion. C. Western blot assay to detect the effects of PELI1 for EMT-related proteins. D. IHC assay detection of the relationship between PELI1 expression and Ki67 expression in PC tissue. **P < 0.01, ***P < 0.001

**Additional file 3: Fig. S3.** FHA Structural Domains are Necessary for PELI1 to Regulate PC Cell Proliferation. A, B, C. CCK-8 (A), clone formation (B), EdU (C) experiments to detect the effect of knockdown FHA structural domains on PC cell proliferation ability. D. Expression of RPS3 mRNA in stably transfected overexpressing or knockdown PELI1 PC cells. *P < 0.05, **P < 0.01, ***P < 0.001, ****P < 0.0001

**Additional file 4: Fig. S4.** Overexpression of RPS3 inhibits PELI1-induced PC proliferation and metastasis. A. Western blot detects changes in cyclin expression after RPS3 overexpression. B, C. The effect of RPS3 on PC cell proliferation and migration was evaluated by EdU assay (B) and wound healing assay (C).*P < 0.05, **P < 0.01, ***P < 0.001, ****P < 0.0001

**Additional file 5: Fig. S5.** PELI1 promotes the proliferation of PC cells through activation of the PI3K/Akt/GSK3β pathway. A. Multiplex immunohistochemical detection of protein expression of PELI1, RPS3, Akt in tumors from subcutaneous tumorigenicity experiments in nude mice. B, C, D. CCK-8 (B), clone formation (C), EdU (D), Experiments were performed to detect the effect of addition of the PI3K inhibitor LY294002 on the proliferation of PC cells. *P < 0.05, **P < 0.01, ***P < 0.001, ****P < 0.0001

**Additional file 6: Fig. S6.** PELI1 promotes p53 degradation in PC cells. A. PELI1 affects p53 polyubiquitination in the K48 and K63 chains. B. Multiplex immunohistochemical detection of protein expression of PELI1, RPS3 and p53 in tumors from subcutaneous tumorigenicity experiments in nude mice

## Data Availability

The original dataset for this study is available by contacting the corresponding author, and all data analyzed in this study are included in the article and additional files.

## Abbreviations

PC: Pancreatic cancer
PDAC: Pancreatic ductal adenocarcinoma
PELI1: Pellino1
RPS3: Ribosomal protein S3
UPS: Ubiquitin-proteasome system
TCGA: The Cancer Genome Atlas
GTEx: Genotype-Tissue Expression
GEO: Gene Expression Omnibus
OS: Overall Survival
PFS: Progression-free survival
DSS: Disease free survival
IHC: immunohistochemistry
IF: Immunofluorescence
EdU: 5-ethynyl-2′-deoxyuridine
CHX: cycloheximide
Co-IP: Co-Immunoprecipitation
