# Supplementary figures and images for: PELI1: key players in the oncogenic characteristics of pancreatic Cancer
Source: J Exp Clin Cancer Res. 2024 Mar 25;43:91. doi: 10.1186/s13046-024-03008-9 (PMC10962118; doi:10.1186/s13046-024-03008-9)

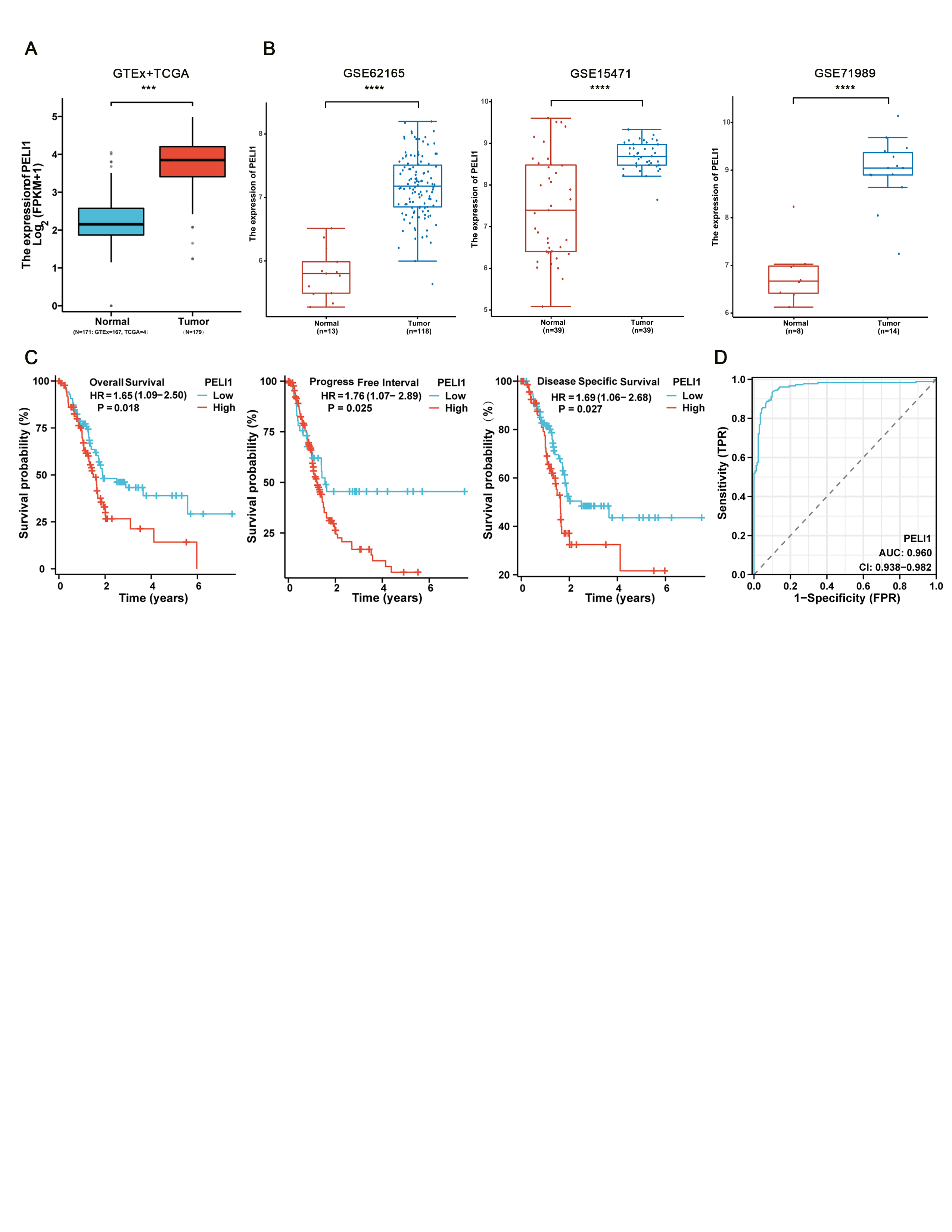

Supplement: Supplementary file 1 — Additional file 1: Fig. S1. Bioinformatics Analysis of the Relationship Between PELI1 Expression in PC Tissues and Prognosis. A. Analysis of PELI1 mRNA expression in PC tissues based on the GTEx and TCGA databases. B. Analysis of PELI1 mRNA expression in PC tissues using three microarray expression datasets (GSE62165, GSE15471, and GSE71989) from the GEO database. C. Association of the high expression of PELI1 with poor prognosis in PC based on the TCGA database. D. ROC analysis demonstrating PELI1's predictive value for the clinical outcome of PC patients. ***P < 0.001, ****P < 0.0001 [file 13046_2024_3008_MOESM1_ESM.tif]

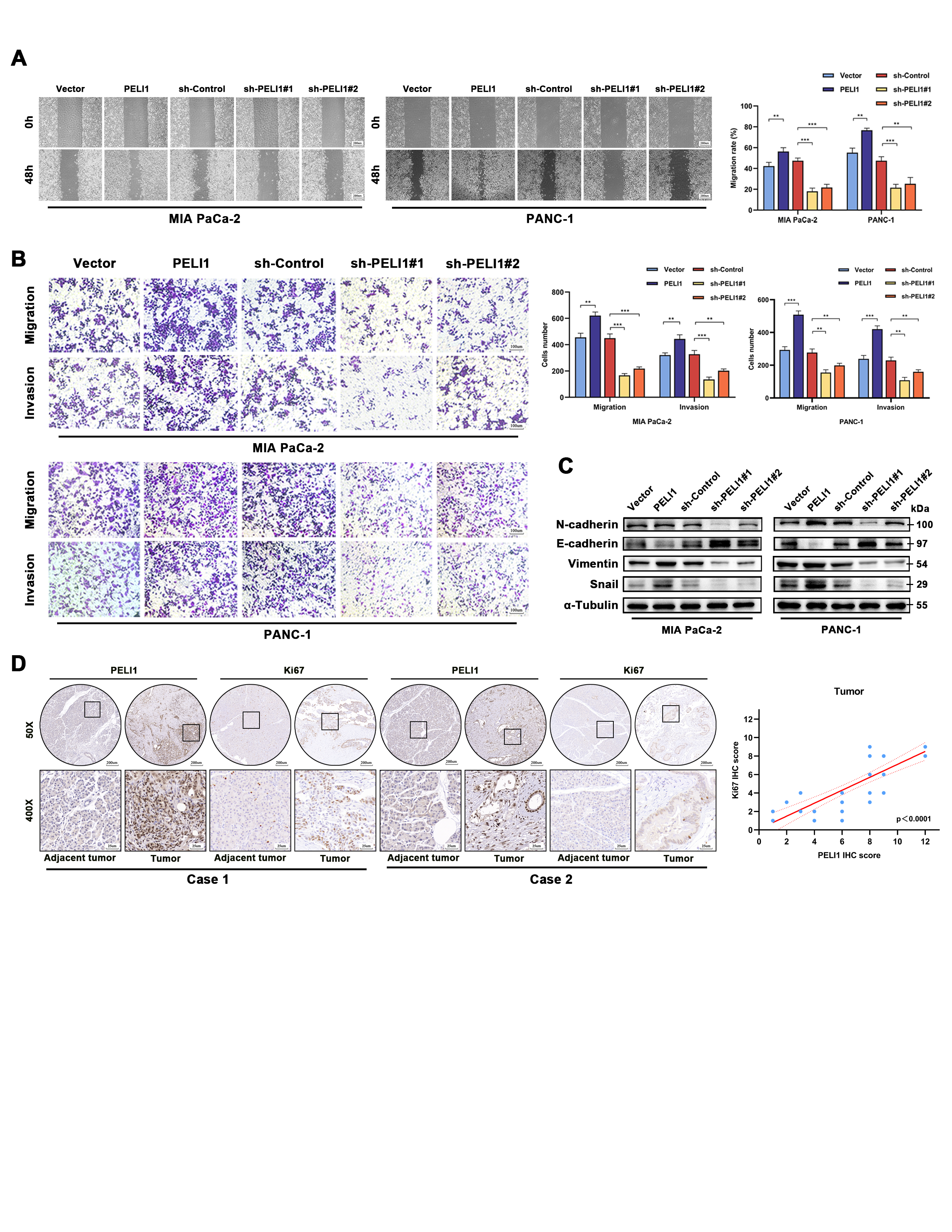

Supplement: Supplementary file 2 — Additional file 2: Fig. S2. PELI1 promotes human PC proliferation, and in vitro invasive and metastatic. A, B Wound healing assay (A) and Transwell assay (B) to detect the effects of PELI1 on PC cell metastasis and invasion. C. Western blot assay to detect the effects of PELI1 for EMT-related proteins. D. IHC assay detection of the relationship between PELI1 expression and Ki67 expression in PC tissue. **P < 0.01, ***P < 0.001 [file 13046_2024_3008_MOESM2_ESM.tif]

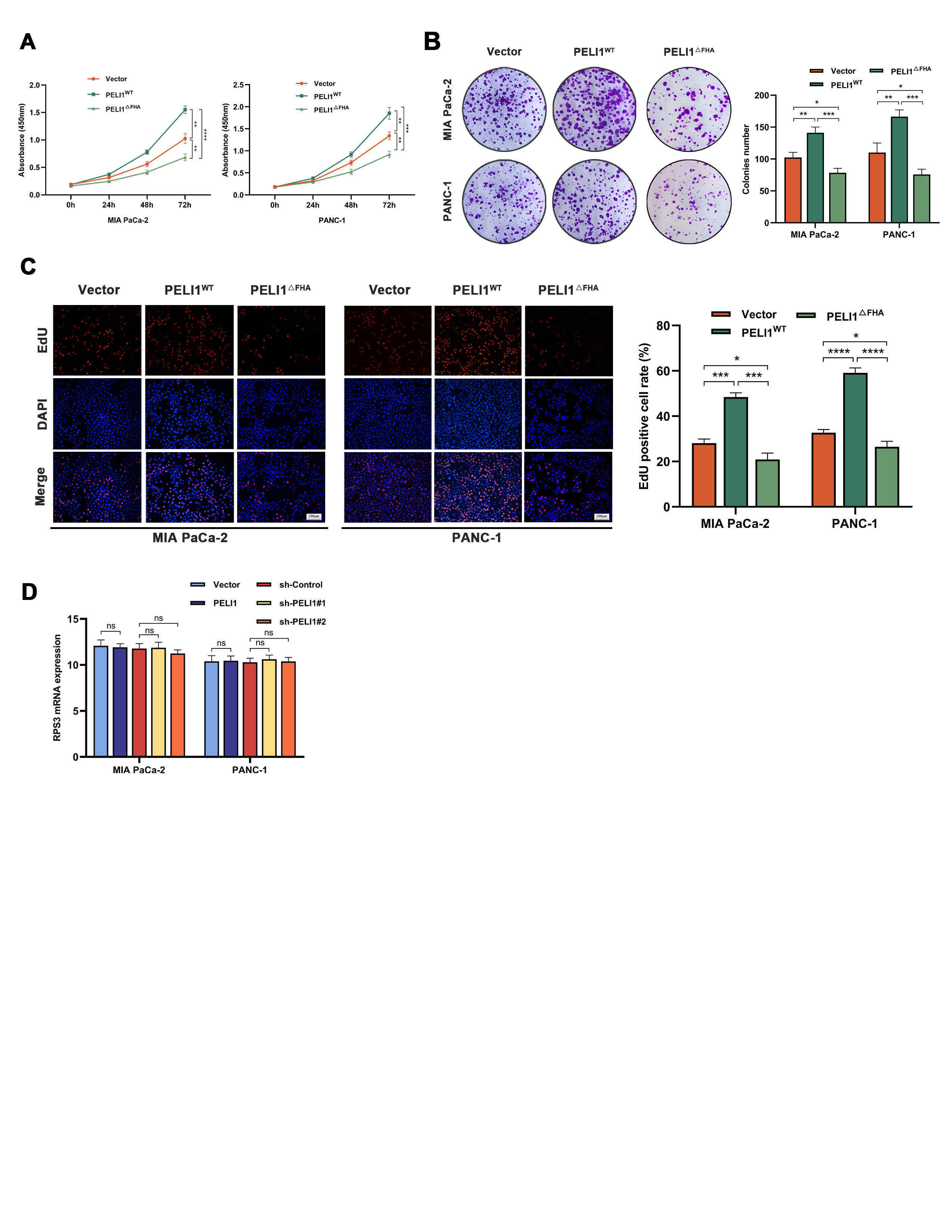

Supplement: Supplementary file 3 — Additional file 3: Fig. S3. FHA Structural Domains are Necessary for PELI1 to Regulate PC Cell Proliferation. A, B, C. CCK-8 (A), clone formation (B), EdU (C) experiments to detect the effect of knockdown FHA structural domains on PC cell proliferation ability. D. Expression of RPS3 mRNA in stably transfected overexpressing or knockdown PELI1 PC cells. *P < 0.05, **P < 0.01, ***P < 0.001, ****P < 0.0001 [file 13046_2024_3008_MOESM3_ESM.tif]

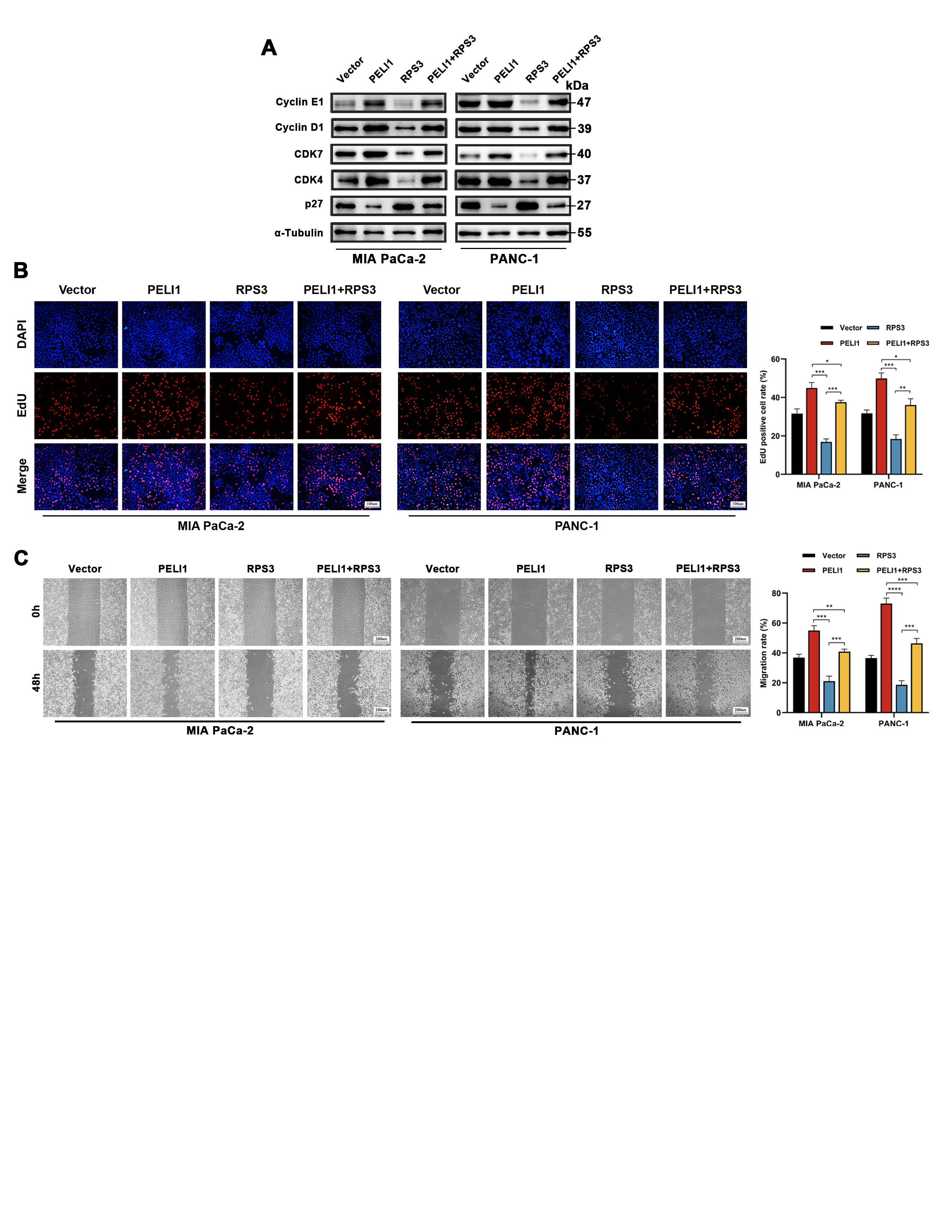

Supplement: Supplementary file 4 — Additional file 4: Fig. S4. Overexpression of RPS3 inhibits PELI1-induced PC proliferation and metastasis. A. Western blot detects changes in cyclin expression after RPS3 overexpression. B, C. The effect of RPS3 on PC cell proliferation and migration was evaluated by EdU assay (B) and wound healing assay (C).*P < 0.05, **P < 0.01, ***P < 0.001, ****P < 0.0001 [file 13046_2024_3008_MOESM4_ESM.tif]

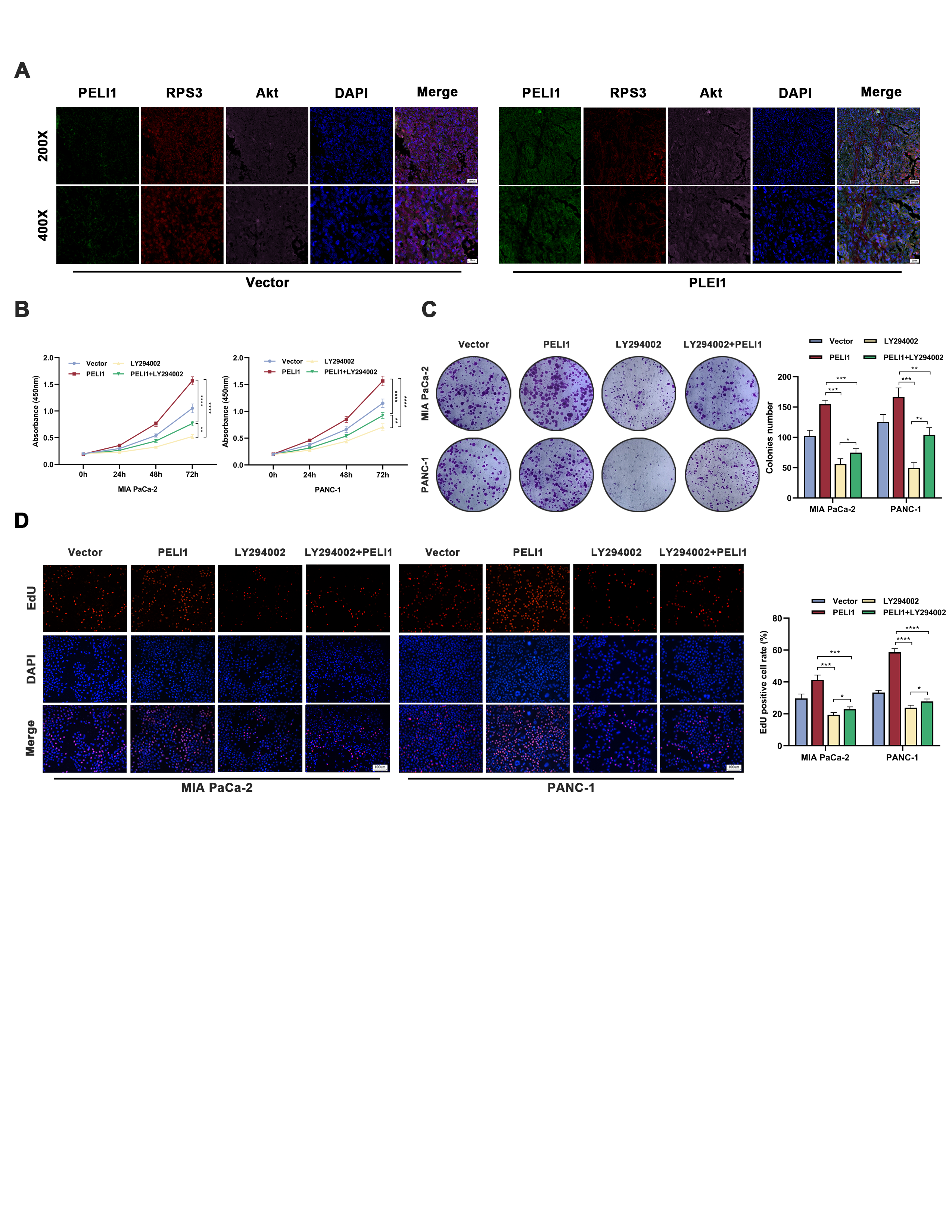

Supplement: Supplementary file 5 — Additional file 5: Fig. S5. PELI1 promotes the proliferation of PC cells through activation of the PI3K/Akt/GSK3β pathway. A. Multiplex immunohistochemical detection of protein expression of PELI1, RPS3, Akt in tumors from subcutaneous tumorigenicity experiments in nude mice. B, C, D. CCK-8 (B), clone formation (C), EdU (D), Experiments were performed to detect the effect of addition of the PI3K inhibitor LY294002 on the proliferation of PC cells. *P < 0.05, **P < 0.01, ***P < 0.001, ****P < 0.0001 [file 13046_2024_3008_MOESM5_ESM.tif]

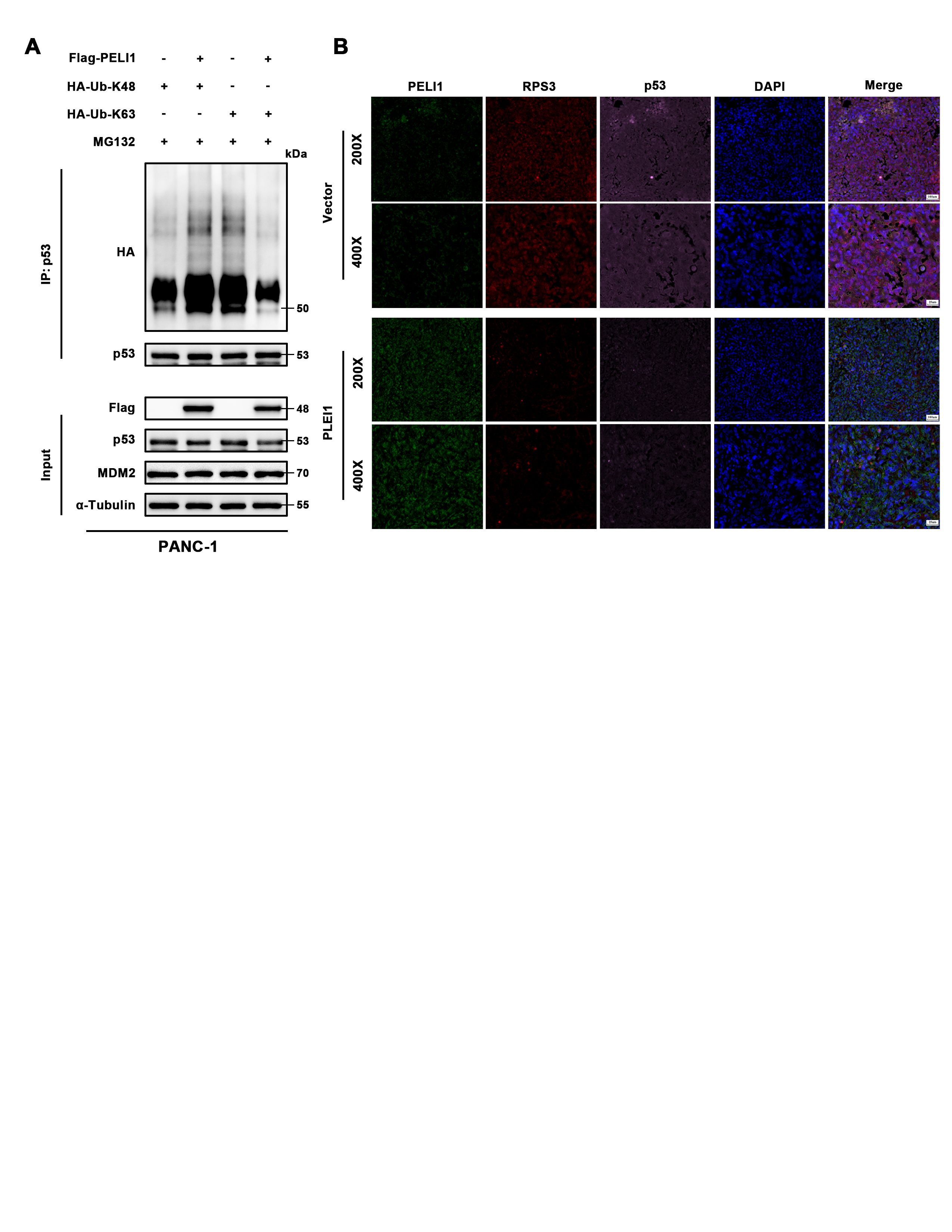

Supplement: Supplementary file 6 — Additional file 6: Fig. S6. PELI1 promotes p53 degradation in PC cells. A. PELI1 affects p53 polyubiquitination in the K48 and K63 chains. B. Multiplex immunohistochemical detection of protein expression of PELI1, RPS3 and p53 in tumors from subcutaneous tumorigenicity experiments in nude mice [file 13046_2024_3008_MOESM6_ESM.tif]
